# Stochastic Simulations Suggest that HIV-1 Survives Close to Its Error Threshold

**DOI:** 10.1371/journal.pcbi.1002684

**Published:** 2012-09-13

**Authors:** Kushal Tripathi, Rajesh Balagam, Nisheeth K. Vishnoi, Narendra M. Dixit

**Affiliations:** 1 Department of Chemical Engineering, Indian Institute of Science, Bangalore, India; 2 Microsoft Research, Bangalore, India; Utrecht University, Netherlands

## Abstract

The use of mutagenic drugs to drive HIV-1 past its error threshold presents a novel intervention strategy, as suggested by the quasispecies theory, that may be less susceptible to failure via viral mutation-induced emergence of drug resistance than current strategies. The error threshold of HIV-1, 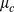, however, is not known. Application of the quasispecies theory to determine 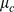 poses significant challenges: Whereas the quasispecies theory considers the asexual reproduction of an infinitely large population of haploid individuals, HIV-1 is diploid, undergoes recombination, and is estimated to have a small effective population size in vivo. We performed population genetics-based stochastic simulations of the within-host evolution of HIV-1 and estimated the structure of the HIV-1 quasispecies and 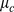. We found that with small mutation rates, the quasispecies was dominated by genomes with few mutations. Upon increasing the mutation rate, a sharp error catastrophe occurred where the quasispecies became delocalized in sequence space. Using parameter values that quantitatively captured data of viral diversification in HIV-1 patients, we estimated 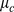 to be 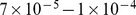 substitutions/site/replication, ∼2–6 fold higher than the natural mutation rate of HIV-1, suggesting that HIV-1 survives close to its error threshold and may be readily susceptible to mutagenic drugs. The latter estimate was weakly dependent on the within-host effective population size of HIV-1. With large population sizes and in the absence of recombination, our simulations converged to the quasispecies theory, bridging the gap between quasispecies theory and population genetics-based approaches to describing HIV-1 evolution. Further, 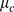 increased with the recombination rate, rendering HIV-1 less susceptible to error catastrophe, thus elucidating an added benefit of recombination to HIV-1. Our estimate of 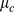 may serve as a quantitative guideline for the use of mutagenic drugs against HIV-1.

## Author Summary

Currently available antiretroviral drugs curtail HIV infection but fail to eradicate the virus. A strategy of intervention radically different from that employed by current drugs has been proposed by the molecular quasispecies theory. The theory predicts that increasing the viral mutation rate beyond a critical value, called the error threshold, would cause a severe loss of genetic information, potentially leading to viral clearance. Several chemical mutagens are now being developed that can increase the mutation rate of HIV-1. Their success depends on reliable estimates of the error threshold of HIV-1, which are currently lacking. The quasispecies theory cannot be applied directly to HIV-1: the theory considers an infinitely large population of asexually reproducing haploid individuals, whereas HIV-1 is diploid, undergoes recombination, and is estimated to have a small effective population size in vivo. We employed detailed stochastic simulations that overcome the limitations of the quasispecies theory and accurately mimic HIV-1 evolution in vivo. With these simulations, we estimated the error threshold of HIV-1 to be ∼2–6-fold higher than its natural mutation rate, suggesting that HIV-1 survives close to its error threshold and may be readily susceptible to mutagenic drugs.

## Introduction

The high mutation rate of HIV-1 coupled with its massive turnover rate in vivo results in the continuous generation of mutant viral genomes that are resistant to administered drugs and can evade host immune responses [1], [2]. The design of drugs and vaccines that exhibit lasting activity against HIV-1 has remained a challenge [3]–[6]. A promising strategy to overcome this challenge has emerged from insights into viral evolution gained from the molecular quasispecies theory [7], [8]. The theory predicts that a collection of closely related but distinct genomes, called the quasispecies, exists in an infected individual when the viral mutation rate is small. When the mutation rate is increased beyond a critical value, called the error threshold, the quasispecies delocalizes in sequence space, inducing a severe loss of genetic information–a phenomenon termed error catastrophe–and compromising the viability of the viral population. It is widely believed therefore that viral mutation rates may have been evolutionarily optimized to lie close to but below their error thresholds so that viral diversity, and hence adaptability, is maximized while genomic identity is maintained [9]–[11]. Consequently, a small increase in the viral mutation rate may trigger an error catastrophe. In accordance, 4-fold increase in the mutation rate induced a dramatic 70% loss of poliovirus infectivity in vitro [9]. Chemical mutagens have been employed successfully to enhance the mutation rates of a host of other viruses [10]–[13] including HIV-1 [14]–[17]. An HIV-1 mutagen is currently under clinical trials [18].

Identification of the host restriction factor APOBEC3G (A3G) has suggested that mutagenesis might also be a natural antiviral defence mechanism (reviewed in [19], [20]). A3G (and, to a smaller extent, APOBEC3F) induces G to A hypermutations in HIV-1, which when unchecked can severely compromise the viability of HIV-1. Interestingly, HIV-1 appears to have evolved a strategy to resist A3G. The HIV-1 protein Vif targets A3G for proteasomal degradation and suppresses its mutagenic activity. Vif thus presents a novel drug target. Inhibiting Vif may enable A3G to exert mutagenic activity adequate to compromise HIV-1. Indeed, significant efforts are underway to develop potent HIV-1 Vif-inhibitors [21].

The use of mutagenesis as an antiviral strategy requires caution because increasing the mutation rate to values below the error threshold could prove counterproductive. The quasispecies theory predicts that a suboptimal increase in the mutation rate would result in an increase in viral diversity that may not be accompanied by a substantial loss of genetic information, which in turn may facilitate the emergence of mutant genomes resistant to drugs and/or host-immune responses [22]. The mutagenic activity of drugs and of host-factors like A3G is dose-dependent [9], [17], [23]. It is important, therefore, to identify the minimum exposure to mutagenic drugs that would ensure that the error threshold of HIV-1 is crossed. The error threshold of HIV-1 is not known.

Translation of the predictions of the quasispecies theory to HIV-1 has remained a challenge: The theory considers the asexual reproduction of a haploid organism with an infinitely large population size, whereas HIV-1 is diploid, undergoes recombination [24]–[27], and is estimated to have a small effective population size in vivo, ∼10^2^–10^5^ cells [28]–[37]. Several studies have advanced the quasispecies theory to account for the diploid nature of HIV-1 and recombination [38]–[50]. The small effective population size of HIV-1 in vivo, however, renders the deterministic formalism of the quasispecies theory inadequate. Population genetics-based stochastic simulations have been resorted to as an alternative [37], [40], [41], [47], [49], [51]–[53]. Such simulations often make significant departures from the quasispecies theory that may render an error catastrophe untenable. For instance, a sharp error catastrophe may not occur with certain fitness landscapes [54]–[58]. Further, in the large population size limit, the simulations may not converge to the predictions of the quasispecies theory [59]. Indeed, whether population genetics- or quasispecies theory-based approaches are more appropriate for describing viral evolution has been the subject of an ongoing debate [56].

We have recently developed stochastic simulations of HIV-1 evolution in vivo that incorporate key aspects of the HIV-1 lifecycle and the underlying evolutionary forces, namely, mutation, multiple infections of cells, recombination, fitness selection using a landscape representative of HIV-1, and random genetic drift [37], [47], [49]. The simulations quantitatively described data of the evolution of viral diversity and divergence in HIV-1 infected individuals over several years following seroconversion, indicating that the simulations faithfully mimicked HIV-1 evolution in vivo [37]. Here, we applied the simulations to determine the structure of the HIV-1 quasispecies and estimate its error threshold. In the limit of large population sizes and in the absence of recombination, our simulations converged to the quasispecies theory, thus bridging the gap between population genetics- and quasispecies theory-based approaches to describing viral evolution and suggesting the existence of an error threshold for HIV-1. We estimated the error threshold of HIV-1 to be ∼2–6-fold higher than its natural mutation rate. HIV-1 thus appears to survive close to its error threshold and may be readily susceptible to mutagenic drugs.

## Results

### Simulations of the within-host evolution of HIV-1

We performed simulations as follows. Uninfected cells were synchronously infected by a pool of identical virions, each cell potentially infected by multiple virions. Viral genomic RNA in cells were then reverse transcribed to proviral DNA. Reverse transcription involved mutation and recombination. The proviral DNA were transcribed to viral genomic RNA, which were assorted into pairs and released as progeny virions. Virions from the pool of progeny virions were selected according to their relative fitness to infect a new generation of uninfected cells, and the cycle was repeated. Following several thousand generations and several such realizations, the expected structure of the viral quasispecies at a given mutation rate was determined. Simulations at different mutation rates allowed identification of the error threshold. Details of the simulation procedure and parameter values employed are presented in Methods.

### Evolution of genome frequencies and the viral quasispecies

**Figure 1:**
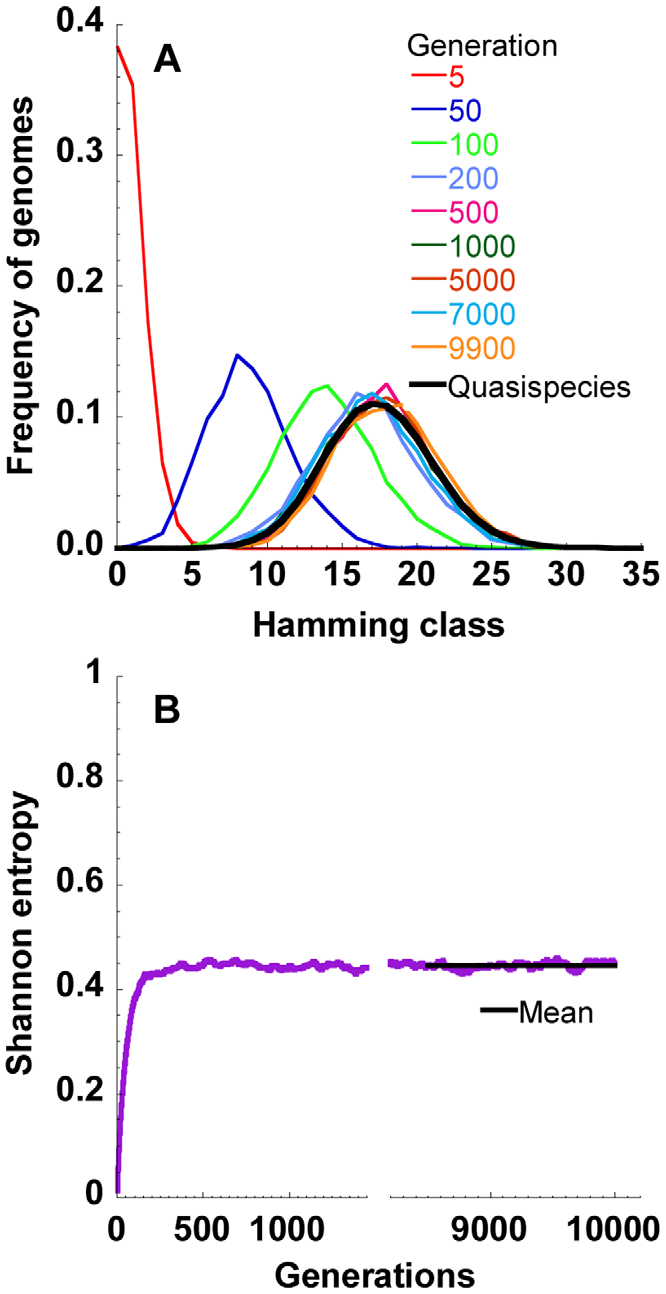
Viral genomic diversification in one realization of our simulations. (A) The frequencies of proviral genomes in different Hamming classes at various times (generations) indicated in one realization of our simulations with 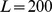 nucleotides, 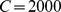 cells, 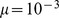 substitutions/site/replication and 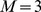 infections/cell. Other parameters are mentioned in Methods. The quasispecies (thick black line) is the average frequency distribution over the last 1500 generations. (B) The corresponding evolution of the Shannon entropy (purple) and its mean over the last 1500 generations (black).

We present first the evolution of the frequencies of genomes in different Hamming classes in one realization of our simulations (Fig. 1). Hamming class 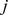 contains genomes carrying 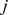 mutations with respect to the fittest, or master, sequence; thus, 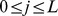, where 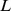 is the genome length. Without loss of generality, we let the fittest sequence be the founder sequence (Fig. S1). Thus, initially, the distribution of genome frequencies was localized at Hamming class zero. As time (or the number of generations) progressed, mutant genomes arose and higher Hamming classes were populated (Fig. 1A). The average number of mutations contained in the proviral pool gradually increased and the peak of the frequency distribution shifted to higher Hamming classes. After a certain number of generations, here ∼500, the distribution became steady; no net shift occurred from generation 500 to 10000. Correspondingly, the Shannon entropy, 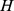, rose from zero at the start and attained the steady value, 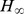, of 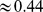 by generation 500 (Fig. 1B). We averaged the above frequencies over the last 1500 generations and over several realizations of our simulations to obtain the expected frequency distribution at steady state. The latter distribution yielded the structure of the viral quasispecies (Fig. 1A).

### Error catastrophe

**Figure 2:**
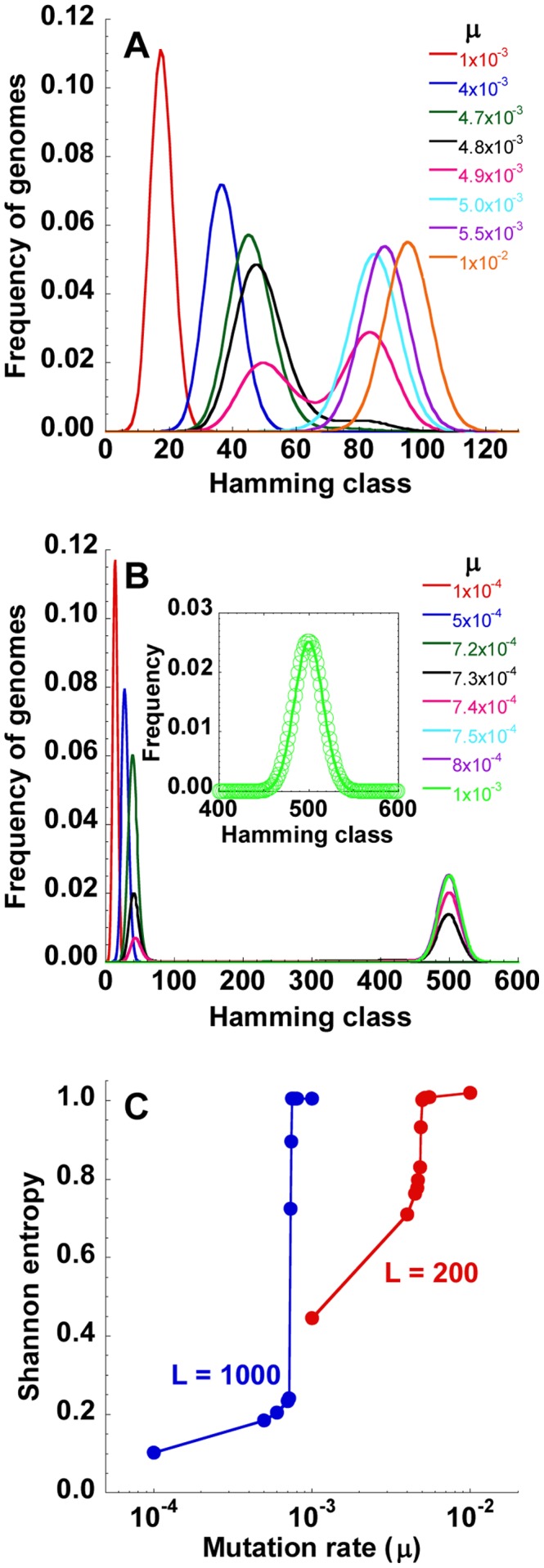
Structure of the quasispecies and the error threshold. The structure of the quasispecies at different values of 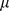 indicated (substitutions/site/replication) with (A) 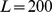 and (B) 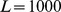 nucleotides. *Inset* in (B) compares the quasispecies structure predicted by our simulations for 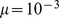 substitutions/site/replication (line) with that expected when all genomes occur with equal likelihood (i.e., 
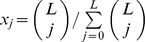
; see text) (symbols). (C) The mean Shannon entropy, 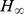, corresponding to the quasispecies in (A) and (B). Other parameters are the same as in Fig. 1.

Upon increasing the mutation rate, 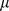, the quasispecies shifted to higher Hamming classes indicating the increasing accumulation of mutations (Fig. 2A). The peak Hamming class (i.e., the Hamming class with the maximum frequency) shifted gradually from 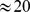 to 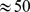 as 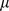 increased from 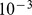 to 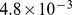 substitutions/site/replication. (Note that 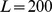 nucleotides in Fig. 2A.) At this point, a small increase in 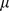 to 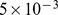 substitutions/site/replication produced a remarkable jump in the peak Hamming class to 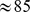. Subsequent increases in 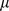 again caused only gradual shifts in the peak Hamming class. This jump was more dramatic with larger genome lengths. With 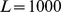 nucleotides, the peak Hamming class jumped from 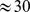 to 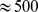 when 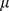 increased from 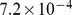 to 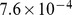 substitutions/site/replication (Fig. 2B). Correspondingly, 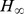 jumped from 0.24 to 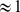 as 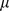 increased from 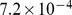 to 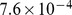 substitutions/site/replication (Fig. 2C). 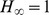 implied that all possible genomes occurred with equal frequencies. The number of distinct genomes in Hamming class 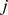 is 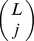. Thus, if all genomes occurred with equal likelihood, the Hamming class frequencies would follow 
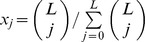
. Indeed, we found that the quasispecies structure obtained by our simulations was identical to the latter distribution of Hamming class frequencies (Fig. 2B inset), confirming that all genomes occurred with equal likelihood when 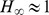. Thus, the jump in 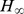 indicated the transition to error catastrophe.

### Error threshold

**Figure 3:**
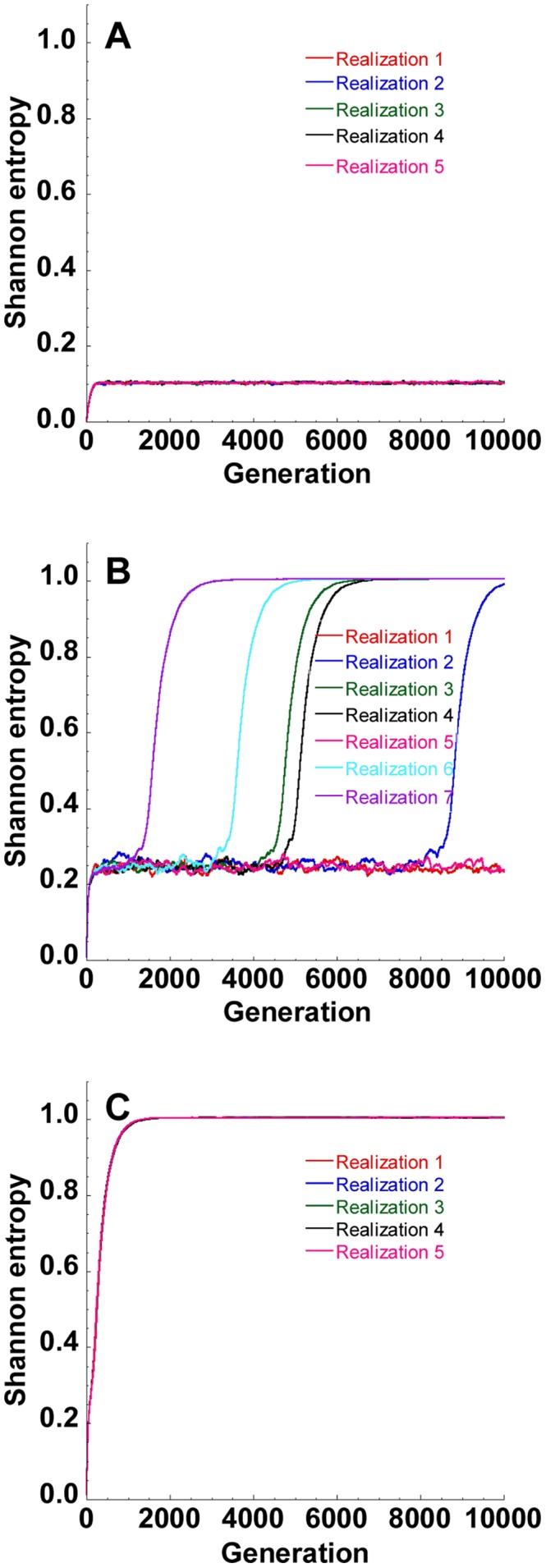
Stochastic evolution near the error threshold. Time-evolution of the Shannon entropy, 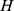, in several independent realizations of our simulations at three values of 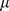, namely, (A) 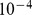, (B) 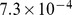 and (C) 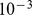 substitutions/site/replication. The other parameters are the same as in Fig. 2B. The different realizations in (A) and (C) nearly overlap and are indistinguishable.

The transition from low 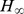 to 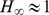 occurred over a narrow range of values of 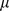. For 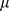 within this range, the quasispecies structure was bimodal because error catastrophe occurred in some realizations and not in others depending on the stochastic variations encountered. For illustration, we present several independent realizations of our simulations at three values of 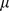, namely, 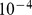, 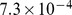 and 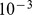 substitutions/site/replication (Fig. 3), where the first is well below the transition from low 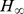 to 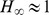, the second is in the transition region, and the third is well above the transition in Fig. 2B. With 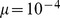 substitutions/site/replication, in each realization 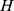 rose from zero and reached 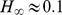 in 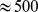 generations (Fig. 3A). There was little variation between the realizations. With 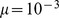 substitutions/site/replication, 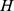 rose from zero and reached 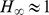 in 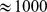 generations, again with little variation between the different realizations (Fig. 3C). With 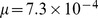 substitutions/site/replication, however, we found substantial variation between realizations (Fig. 3B). 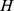 rose from zero and reached a plateau value of 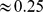 in 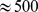 generations. In some realizations, 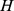 remained at this value till the end, i.e., 10000 generations. In other realizations, at some intermediate time, which differed from realization to realization, 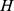 rose sharply from 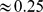 and reached 1. 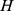 remained at 1 subsequently. Averaging the Hamming class frequencies thus yielded the bimodal structure of the quasispecies observed for 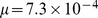 substitutions/site/replication (Fig. 2B), where realizations with 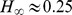 yielded the peak at Hamming class 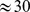 and realizations with 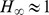 yielded the peak at Hamming class 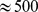.

Our aim was to identify the smallest value of 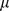 at which error catastrophe was ensured. We found that when 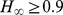 stochastic variations became insignificant and error catastrophe occurred nearly invariably. We therefore identified the smallest 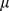 for which 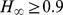 as the error threshold, 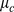. Thus, 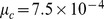 and 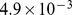 substitutions/site/replication for 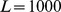 and 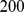 nucleotides, respectively, in Fig. 2C.

### Influence of model parameters on the error threshold

#### Genome length

**Figure 4:**
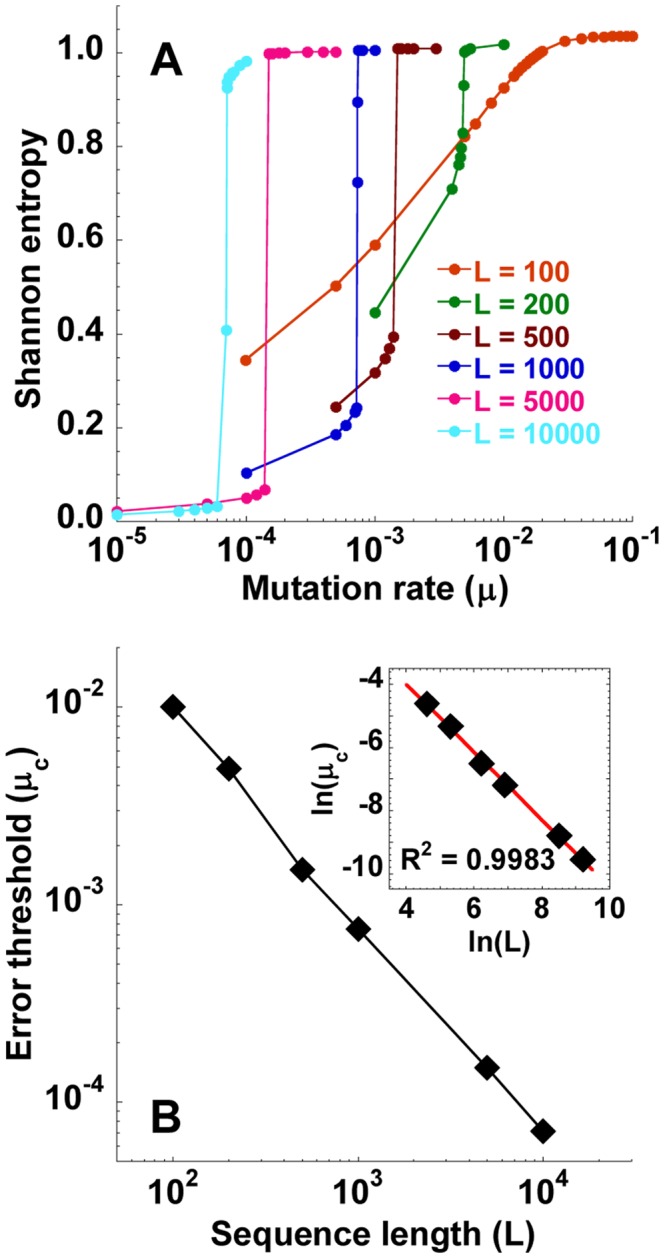
Dependence of the error threshold on the genome length. (A) The mean steady state Shannon entropy, 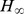, as a function of the mutation rate, 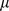, for different genome lengths, *L* (nucleotides), indicated. Other parameters are the same as in Fig. 1. The corresponding structures of the quasispecies are shown in Fig. S2. (B) The resulting dependence of the error threshold, 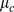, on *L*. *Inset* in (B) shows a linear fit (line) to the data (symbols) yielding 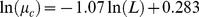.

Upon increasing 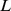, the transition to error catastrophe became sharper and occurred at lower values of 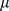 (Fig. 4). For instance, 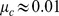 substitutions/site/replication when 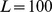 nucleotides and 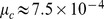 substitutions/site/replication when 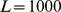 nucleotides (Fig. 4A). Further, 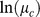 decreased linearly with 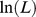 with a slope of −1.07 (Fig. 4B) indicating that 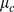 is approximately proportional to 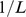. These predictions that the transition sharpens with increasing 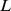 and that 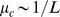 are in agreement with the quasispecies theory [7], [8].

#### Population size

**Figure 5:**
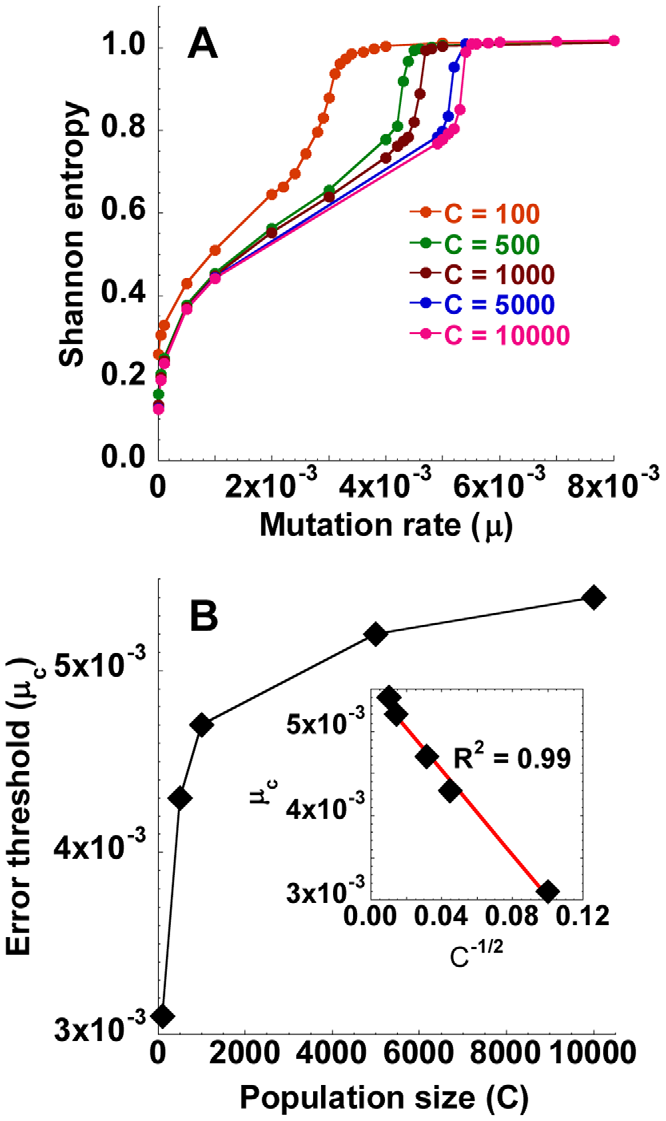
Dependence of the error threshold on the population size. (A) The mean steady state Shannon entropy, 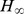, as a function of the mutation rate, 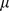, for different population sizes, *C* (cells), indicated. Other parameters are the same as in Fig. 1. The corresponding structures of the quasispecies are shown in Fig. S3. (B) The resulting dependence of the error threshold, 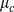, on *C*. *Inset* in (B) shows a linear fit (line) to the data (symbols) yielding 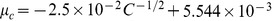.

Increasing the cell population, 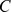, increased 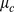 (Fig. 5). For instance, 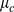 increased from 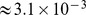 to 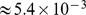 substitutions/site/replication as 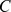 rose from 100 to 10000 cells (Fig. 5A). Eventually, the dependence of 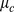 on 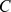 weakened and 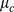 appeared to plateau asymptotically as 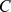 increased (Fig. 5B). Further, 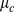 decreased linearly as 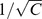 increased (Fig. 5B inset), as suggested by previous extensions of the quasispecies theory to finite populations [59], [60]. A fit to the data in Fig. 5B yielded 
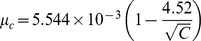
 (Fig. 5B inset). Extrapolation provided an estimate of 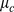 for 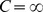, which for the parameters in Fig. 5B was 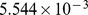 substitutions/site/replication.

#### Recombination and multiple infections of cells

**Figure 6:**
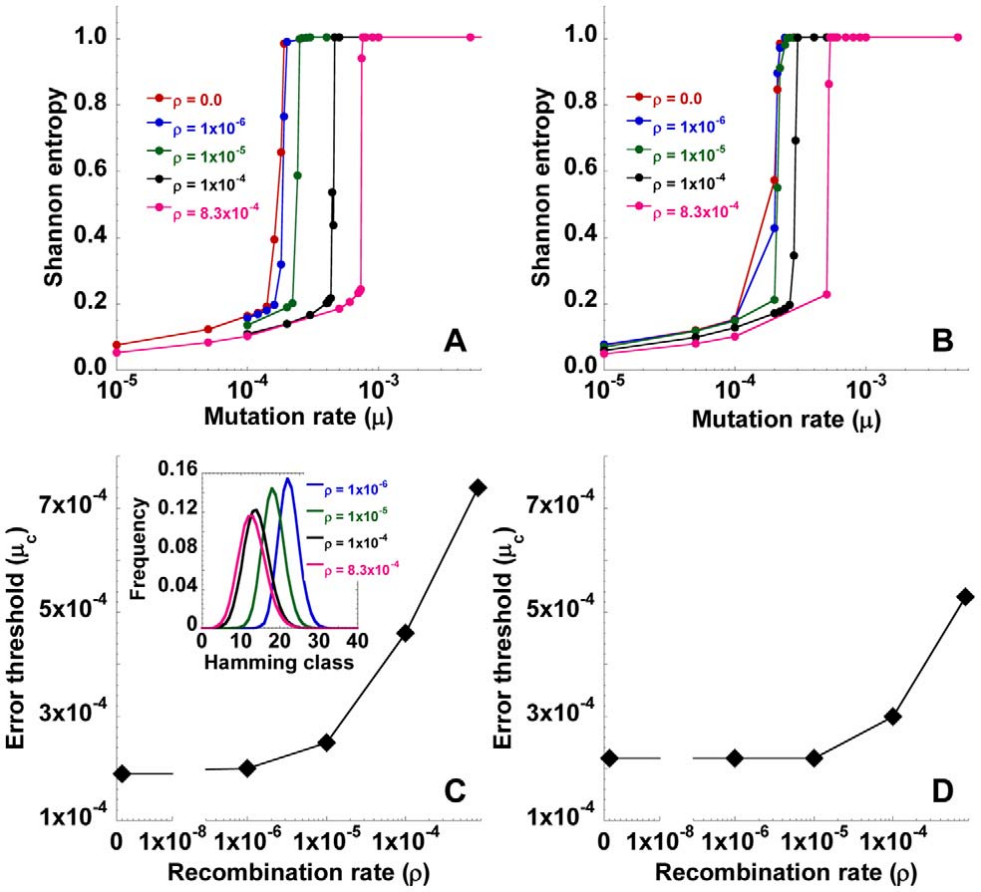
Dependence of the error threshold on the recombination rate. The mean steady state Shannon entropy, 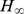, as a function of the mutation rate, 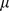, for different recombination rates, 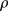, indicated (crossovers/site/replication) with (A) 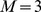 infections/cell and (B) 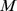 determined from a distribution with few multiple infections (Methods). Here, 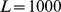 nucleotides, 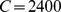 cells, and the other parameters are the same as in Fig. 1. The corresponding structures of the quasispecies are shown in Fig. S4 and Fig. S5, respectively. (C) and (D) The resulting dependence of the error threshold, 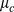, on 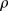 in (A) and (B), respectively. *Inset* in (C) shows the quasispecies for different 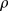 (crossovers/site/replication) indicated with 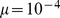 substitutions/site/replication and 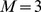 infections/cell.

Increasing the recombination rate, 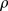, or the number of infections per cell, 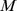, also increased 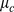 (Fig. 6). 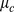 rose from 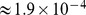 to 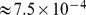 substitutions/site/replication as 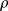 increased from zero to 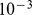 crossovers/site/replication when 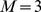 infections/cell (Fig. 6A and C). Similarly, 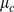 rose from 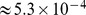 to 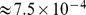 substitutions/site/replication as 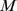 increased from 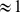 infection/cell, following a distribution with few multiple infections, to 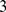 infections/cell when 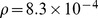 crossovers/site/replication (Fig. 6B, C, and D). The quasispecies structure at fixed 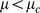 shifted to smaller peak Hamming classes and widened upon increasing 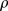 (Fig. 6C inset), consistent with our previous observations that recombination increased the mean fitness and the diversity of the quasispecies with small 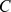 [47] (also see Discussion). Consequently, higher mutation rates were necessary to induce an error catastrophe as 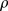 increased. Increasing 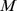 effectively increased recombination [51], [61] and hence also resulted in an increase in 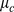.

#### Mutation and genome sequence composition

The HIV-1 genome is known to be A rich [62]. Besides, not all mutations occur at the same rate; G to A transitions are the most frequent [63]. We therefore performed simulations with a founder sequence containing nucleotides at frequencies corresponding to those in HIV-1 and with nucleotide-specific transition rates mimicking HIV-1 (Methods). When all nucleotides were equally represented but mutations occurred in a nucleotide-specific manner, we found that 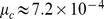 substitutions/site/replication (Fig. S6), which is close to 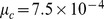 substitutions/site/replication when mutations occurred in a nucleotide independent manner (Fig. 2). Further, 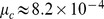 substitutions/site/replication when the founder sequence mimicked the HIV-1 nucleotide frequencies and mutations occurred in a nucleotide-specific manner (Fig. S6). Thus, 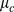 did not depend significantly on the nucleotide composition of the founder sequence (as also observed in Fig. S1) and on whether mutations occurred in a nucleotide-independent manner or the observed nucleotide-specific manner.

#### Fitness landscape

The above simulations employed a fitness landscape derived from data [64] of in vitro replicative fitness assays (see [37] and Methods). To examine whether our predictions were specific to the fitness landscape employed, we performed simulations with several alternative theoretical landscapes. First, we modified our present landscape to allow genomes to have zero fitness: we set the fitness of all genomes below a particular threshold, 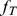, in the above landscape to zero (Methods). The resulting landscape is similar to the truncated landscape employed previously (e.g., see [65]). The minimum fitness in the above landscape was 0.24. We performed simulations with 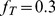 and 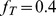 with 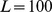 nucleotides and found little variation in 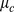 from the above estimate (Fig. S7A) (also see Discussion). In a previous study, we found that an exponential fitness landscape, which assigns a fixed fitness penalty for every mutation (see below), does not agree with patient data and thus may not be representative of HIV-1 in vivo [37].

The complex fitness interactions of HIV-1 mutations unraveled recently [66] have been characterized using a fitness landscape that accounts sequentially for the effects of individual mutations, interactions between pairs of mutations, between triplets of mutations, and so on [67], akin to spin glass-based and other correlated landscapes employed earlier [60]. We found that such a landscape reduced under limiting conditions to a polynomial in the Hamming distance of genomes from the master sequence (Methods). We identified the coefficients of the polynomial by fitting mean fitness data (Fig. S7B inset) and performed simulations with the resulting best-fit polynomial landscape. We found that 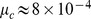 substitutions/site/replication (Fig. S7B), close to 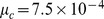 substitutions/site/replication obtained with the landscape above (Fig. 2), indicating only a minor influence of these modifications to the fitness landscape on 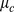.

### Comparison with quasispecies theory

**Figure 7:**
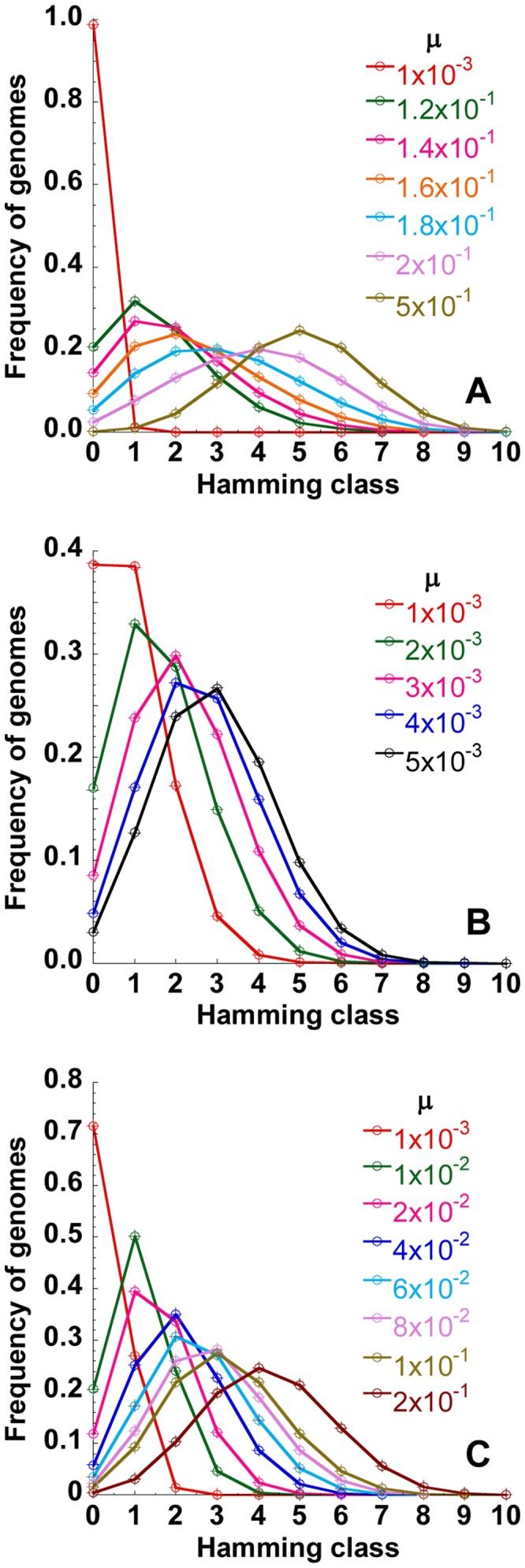
Comparisons of our simulations with the quasispecies theory. Structure of the quasispecies for different values of 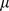 (substitutions/site/replication) indicated determined by our simulations (circles connected by lines) and by the quasispecies theory (pluses) for (A) isolated peak fitness landscape, (B) exponential landscape with *s* = 0.01, and (C) the experimental landscape with *d_50_* = 3.

To test whether our simulations converged to the quasispecies theory, we performed simulations with parameter values that mimic the assumptions employed in the quasispecies theory. We let 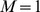 infection/cell and 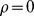 crossovers/site/replication to represent the asexual reproduction of effectively haploid individuals. We chose a large population size, 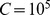 cells, and a small genome length, 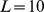 nucleotides, to approximate the infinite population size limit (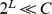). We employed the single peak fitness landscape, typically employed in calculations of the quasispecies theory, which we implemented by letting viral production be 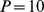 virions/cell for cells infected with the master sequence and 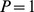 virion/cell for all other cells and then selecting virions with equal probability from the viral pool. We also solved the equations of the quasispecies theory using the latter fitness landscape (Methods). Remarkably, our simulations were in excellent agreement with the quasispecies theory for a wide range of mutation rates (Fig. 7A).

To test the robustness of this agreement, we performed simulations with two other fitness landscapes, an exponential landscape, 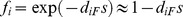, where the relative fitness declined nearly linearly (at rate 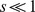 per mutation) with the number of mutations from the master sequence, 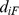, and the experimental landscape above rescaled to the smaller genome length. In both these cases, we let 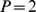 virions/cell in our simulations and selected virions in proportion to their relative fitness. Again, our simulations were in excellent agreement with solutions of the quasispecies theory using the latter fitness landscapes (Figs. 7B and C).

Thus, with large population sizes, our simulations were in quantitative agreement with the quasispecies theory. With smaller population sizes, our simulations predicted trends that were consistent with previous finite population models of genomic evolution. Further, with parameter values representative of HIV-1 infection in vivo, we showed previously that our simulations quantitatively described patient data of the evolution of viral diversity and divergence over extended durations (∼10–12 years) [37], giving us confidence in our simulations. We employed our simulations to estimate the error threshold of HIV-1.

### Estimate of the error threshold of HIV-1

**Figure 8:**
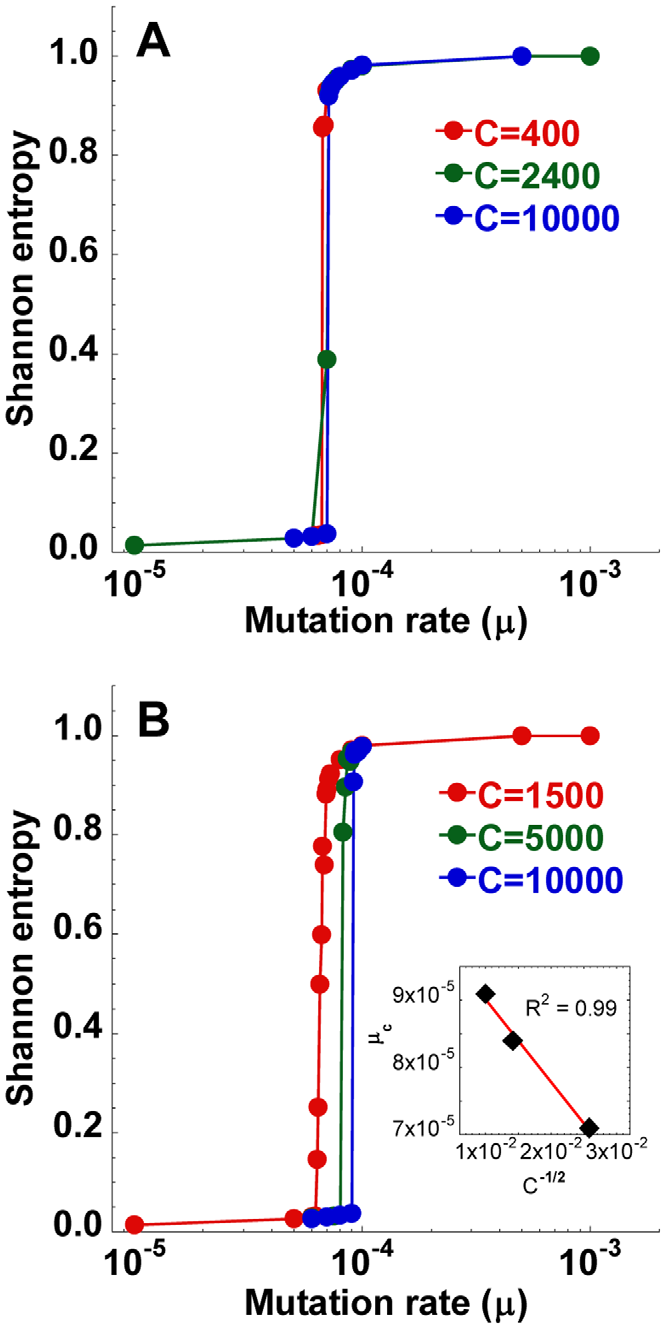
Estimates of the error threshold of HIV-1. Mean steady state Shannon entropy, 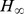, as a function of the mutation rate, 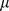, for different population sizes, 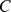 (cells), indicated with (A) 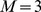 infections/cell and (B) 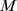 determined from a distribution with few multiple infections of cells, where 77% of the cells were singly, 19% doubly, and 4% triply infected. Other parameters are as follows: 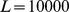 nucleotides; 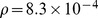 crossovers/site/replication; 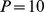 infectious rogeny virions/cell; the fitness landscape 
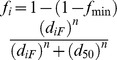
, where 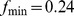, 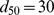, and 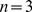 (Methods). The corresponding structures of the quasispecies are shown in Fig. S8 and Fig. S9, respectively. *Inset* in (B) shows a linear fit (line) to the data (symbols) yielding 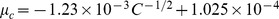.

We performed simulations with parameter values that mimic patient data of viral genomic diversification quantitatively (Methods). We previously analyzed data of viral diversity and divergence from 9 patients [68] and found that with 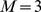 infections/cell, following observations of Jung et al. [24], the best-fit values of 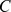 varied from 400–10000 cells across the patients with a mean of 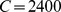 cells [37]. Accordingly, we performed simulations here with 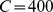, 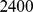, and 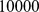 cells. We found a sharp error catastrophe with 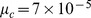, 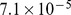 and 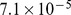 substitutions/site/replication, respectively (Fig. 8A). A smaller frequency of multiple infections of cells, mimicking the observations of Josefsson et al. [69], was also able to capture the same patient data with higher best-fit values of 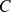 [37]. Then, except for one patient (Patient 11), for whom 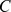 was 10^5^ cells, the best-fit values of 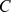 were in the range of 1500–10000 cells. Recognizing that the dependence of 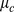 on 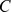 was weak for large 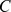, we performed simulations with 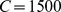, 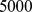, 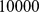 cells (where 5000 cells was the mean for the remaining 8 patients) using 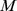 drawn from a distribution mimicking the observations of Josefsson et al. We found again that a sharp error catastrophe occurred with 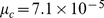, 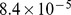 and 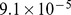 substitutions/site/replication for the three cases (Fig. 8B), close to the estimates above. The modest increase of 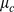 with 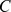 again displayed the 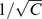 dependence (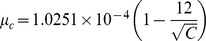, Fig. 8B inset) and yielded 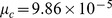 substitutions/site/replication for 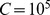 cells and 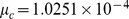 substitutions/site/replication for 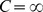. Taken together, our simulations predict that HIV-1 undergoes a sharp error catastrophe and estimate the error threshold to be in the range 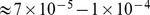 substitutions/site/replication.

## Discussion

The success of mutagenic drugs against HIV-1 hinges on reliable estimates of the error threshold of HIV-1, which are currently lacking. The assumptions employed in the quasispecies theory render it inadequate for describing HIV-1 evolution. Here, we have employed population genetics-based simulations of HIV-1 evolution to examine the susceptibility of HIV-1 to mutation-driven error catastrophe. With these simulations, we found that HIV-1 experienced a sharp error catastrophe at a mutation rate of 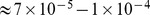 substitutions/site/replication. Our simulations incorporated key evolutionary forces underlying the within-host genomic diversification of HIV-1 and were shown previously to be in agreement with longitudinal patient data of viral diversity and divergence [37], giving us confidence in our estimate of the error threshold. That the estimated error threshold is ∼2–6 fold higher than the natural mutation rate of HIV-1 in vivo, 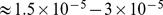 substitutions/site/replication [63], [70], suggests that HIV-1 exists close to its error threshold. The mutation rate of HIV-1 thus appears to be evolutionarily optimized to maximize diversity while retaining genomic identity. A relatively small (2–6 fold) increase in the mutation rate may thus drive HIV-1 past its error threshold, presenting a quantitative guideline for mutagenic drugs.

The quasispecies theory has presented remarkable insights into viral evolution and suggested new strategies of intervention [9], [71]–[74]. Yet, its ability to describe viral evolution comprehensively is limited, as recognized by Eigen himself [75], by its assumptions of, for instance, an infinitely large population size, asexual reproduction of haploid organisms, and an isolated peak fitness landscape where all mutants are equally less fit than the master sequence. The last 40 years have seen significant efforts to relax these assumptions and tailor the quasispecies theory to specific organisms, especially HIV: Several, more complex and more realistic fitness landscapes have been employed [37], [49], [54], [55], [58], [60], [76]–[78]. Simultaneously, population genetics-based approaches, which naturally consider stochastic effects associated with finite populations, have been developed [59], [60], [79]–[85]. The latter descriptions, however, while painting a more realistic picture of the organisms considered, often make marked deviations from the key predictions of the quasispecies theory. In particular, finite population models may not converge to the quasispecies theory in the infinite population limit [59], or with more complex fitness landscapes, a sharp error catastrophe may cease to occur [54]–[58]. Consequently, questions arise of the relative merits and appropriateness of using the quasispecies theory or population genetics-based approaches to describe viral evolution (reviewed in [56]). Here, we showed that our simulations converge to the quasispecies theory in the large population size limit, indicating that quasispecies theory is not at odds with population genetics-based descriptions at least of HIV-1. In a related study, convergence of similar population genetics-based descriptions to the quasispecies theory has been established formally [86]. Importantly, with a fitness landscape representative of HIV-1 [64], and with other parameters that mimic patient data, our simulations predict that a sharp error threshold exists for HIV-1.

In our simulations, the error threshold scaled linearly with 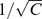, where *C* is the population size of cells, in agreement with previous studies [59], [60]. We note that some studies using alternative simulation strategies found a linear scaling with 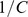 [80]. The origin of this discrepancy in the dependence of the error threshold on *C* remains to be established. Nonetheless, the weak dependence of the error threshold on *C* implies that our estimate of the error threshold remains robust to any increase in the effective population size in vivo either due to inter-patient variations or due to uncertainties in the estimates of model parameters. We showed previously that estimates of the effective population size of HIV-1 in vivo were sensitive to the frequency of multiple infections of cells, *M*, and the recombination rate [37]. Few estimates of *M* in vivo are available. While one study of infected splenocytes in two patients found that most cells were multiply infected with a mean of 3–4 proviruses per cell [24], recent evidence from peripheral blood mononuclear cells of several acute and chronically infected individuals suggests that multiple infections of cells may be rare [69], and hence the influence of recombination weak [51], [61]. Using parameters corresponding to either observation, we found that our simulations captured patient data of viral diversification with appropriate values of *C* [37]. Using both combinations of *M* and *C* that matched patient data, we estimated the error threshold of HIV-1 here and found that the estimates were close, suggesting that uncertainties in the frequency of multiple infections did not significantly affect our estimate of the error threshold.

The role of recombination in HIV-1 evolution has remained difficult to interpret [2], [39], [87]. Just as recombination can bring favorable mutations together, it can also drive favorable combinations of mutations apart, raising questions more generally about the evolutionary origins of the ubiquitously present recombination and sexual reproduction, often referred to as the paradox of sex [39], [88], [89]. The benefit of sex has recently been suggested to arise from the subtle interactions of random genetic drift, selection, and recombination in finite populations [90]. When the population size is small, negative linkage disequilibrium (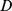) is generated by the Hill-Robertson effect [91]. Recombination lowers the absolute value (magnitude) of 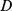, which when 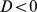 enhances diversity and favors selection [89], [92]–[94]. Indeed, our simulations showed that as the recombination rate increased, the quasispecies shifted to lower peak Hamming classes and spread wider, implying greater average fitness and diversity. In agreement, we showed previously that the mean fitness and diversity of the viral population increased with recombination when the population size was small [47]. An added advantage of recombination that we found here was that the error threshold also increased with recombination, rendering the quasispecies more resistant to mutation-driven loss of genetic information. In an earlier study, recombination was found in contrast to decrease the error threshold [38]. The latter study, however, considered an infinitely large population size with a single peak landscape, which is expected to generate 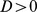. Accordingly, the lowering of 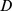 by recombination decreases diversity and is therefore expected to lower the error threshold. 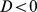 generated by the Hill-Robertson effect underlies the enhancement of the error threshold due to recombination in our simulations. Given that host factors such as A3G combat HIV-1 by increasing the viral mutation rate [19], [20], recombination, in synergy with Vif-induced degradation of A3G, may serve to stall the onslaught of A3G and establish lasting infection.

The population sizes we employed were obtained by fits of our simulations to patient data [37]. The census population size of HIV-1 is ∼10^7^–10^8^ infected cells [95]. Yet, the effective population sizes obtained by several independent studies are small and lie in the range of ∼10^2^–10^5^ cells (reviewed in [35]). The effective population size is defined as the size of the population in an idealized model of evolution that has the same population genetic properties as that of the natural population [96]. The reasons underlying the differences between the census and effective population sizes of HIV-1 remain to be established; bottlenecks introduced by the immune system and other selection pressures [36], asynchronous infections of cells [97], pseudohitchhiking [98], and metapopulation structure [99] may all contribute to the small effective population sizes estimated, but their roles in HIV-1 evolution are yet to be fully elucidated. We employed a fitness landscape that is a measure of the relative replicative ability of various HIV-1 mutants determined using in vitro assays [64]. The landscape suggests that the predominant fitness effects depend on the number and not on the specific combinations of mutations, allowing us to group genomes into Hamming classes [100]. Simpler fitness landscapes, such as multiplicative landscapes, were not compatible with patient data [37]. More comprehensive fitness interactions are beginning to be unraveled [66], [101]. The resulting fitness values [66] have been shown to be correlated with the viral load in vivo [102]. Under certain limiting conditions, we found that the latter interactions yielded a fitness landscape consistent with the landscape we employed above (Methods and Fig. S7). Further, our estimates of the error threshold were robust to minor variations in the fitness landscape. For instance, allowing lethal mutations using a truncated landscape, where genomes with fitness below a certain threshold were assumed replication incompetent, did not substantially alter the error threshold (Fig. S7). We recognize that lethal mutations can occur more frequently; for instance, 40% of random mutations in an RNA viral genome were found to be lethal [103]. Such a scenario is estimated to increase the error threshold for an infinitely large population size and a single peak fitness landscape by a factor of ∼5/3 [104], [105]. Understanding the influence of major variations in the fitness landscape is computationally prohibitive and awaits future studies. Finally, we recognize that we have assumed uniform recombination rates and either uniform or nucleotide-specific mutation rates across the HIV-1 genome, whereas mutation [63] and recombination hot-spots [106], [107] are known to exist within HIV-1.

Estimation of the error threshold of HIV-1 from experimental studies of viral mutagenesis-induced loss of viral infectivity has not been possible because of several confounding effects. For instance, 2–3 fold increase in the mutation rate obliterated HIV-1 infection in vitro [14], [15], in agreement with our present findings. The agreement, however, is not conclusive because establishing that the loss of infectivity in vitro is due to an error catastrophe is not straight-forward. The loss of infectivity may be due to an error catastrophe, as demonstrated with poliovirus [9], but may also arise from other effects: At mutation rates above the natural mutation rate but below the error threshold, production of defective genomes may drain resources within cells, compromising the production of viable genomes and causing extinction of the viral population [13]. Thus, whether viral extinction necessarily implies crossing the error threshold remains unclear. Conversely, crossing the error threshold may not imply viral extinction; the latter may require crossing an alternative ‘extinction’ threshold, where each viral particle produces less than one progeny that infects a cell, akin to the epidemiological threshold for extinction of disease [108]. (Note that in our present simulations, infection was sustained by keeping the pool of infected cells constant.) Viral extinction may also be determined by the influence of mutations on protein stability and its impact on viability [109], [110]. Establishing which of these phenomena underlies the observed loss of viral infectivity in vitro remains a challenge.

Finally, we recognize that the dynamics of the transition to error catastrophe, which remains poorly characterized, is also of importance to mutagenic strategies targeting HIV-1. For instance, 9–24 serial passages were required for loss of viral infectivity in vitro [14]. In a recent clinical trial with an HIV-1 mutagen, no viral load decline was observed in patients following 124 days of treatment although the mutational patterns were altered [18]. This absence of apparent antiviral activity was attributed to the lack of knowledge of both the level and the duration of exposure of the drug necessary to compromise the viability of HIV-1 [18], reiterating the importance of reliable estimates of the error threshold and of the timescales of the transition. Our estimate of the error threshold together with the dose-response data of the drug may help determine the level of drug necessary to induce an error catastrophe in HIV-1. Further, although we focused here on identifying the structure of the HIV-1 quasispecies and estimating its error threshold, our simulations present a framework for determining the time required to ensure completion of the transition to error catastrophe, thus elucidating guidelines for the duration of treatment with mutagenic drugs.

## Methods

### Simulation protocol

#### Creation of the viral pool

We represented an HIV-1 genome as a sequence of 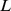 nucleotides. We generated such a sequence with each nucleotide chosen randomly from A, G, C and U with equal probability or with probabilities representative of the nucleotide content in HIV-1 (see below). We let the resulting sequence be the master sequence and set its relative fitness to unity. We represented a virion by the pair of RNA genomes it contained. We let the initial pool of 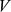 virions all carry the master sequence.

#### Infection of cells

We considered a pool of 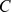 uninfected cells. We randomly selected 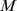 virions, each with a probability equal to its relative fitness (see below), from the viral pool to infect one of the uninfected cells. 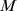 was either constant or drawn from a predetermined distribution (see below). The genomes of the chosen virions were transferred to the cell and the virions were removed from the viral pool. This process was repeated for each of the remaining cells.

#### Reverse transcription

Following infection, the viral RNA were mutated and recombined. We considered one of the 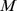 pairs of viral RNA within an infected cell. We selected one of the two genomes in the pair randomly and began copying its nucleotide sequence bit by bit to the resulting recombinant DNA genome. At each position, we switched templates to the other RNA strand with probability 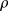, the recombination rate, thus producing a recombinant genome that was a mosaic of the two parent viral RNA genomes. Next, we mutated the recombinant genome with probability 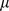 at every position, where 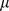 was the mutation rate. In some simulations, we let the probability be nucleotide-specific (see below). The resulting sequence was the proviral DNA produced by reverse transcription. We repeated this process for the remaining pairs of viral RNA within the cell and in all the other cells.

#### Viral production

Each infected cell produced 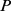 progeny virions. For each virion produced from a cell, we randomly chose two of the 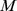 proviral DNA present in the cell and assigned their sequences as the viral RNA genomes of the virion. When *M* = 1, the same provirus was chosen twice. The transcription of proviral DNA to viral RNA is catalyzed by host proteins and introduces far fewer mutations than reverse transcription. We therefore assumed that no mutations occurred during proviral DNA transcription. The resulting progeny virions constituted the new viral pool for infecting the next generation of uninfected cells.

We repeated the process for a large number of generations (see below) and averaged over many such realizations to obtain the expected evolution for a given set of parameter values. We performed the simulations using a computer program written in C++ (Text S1).

### Measures of viral evolution and quasispecies structure

#### Hamming class frequencies

In each generation we determined the number of proviral genomes, 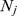, belonging to different Hamming classes, 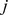, where 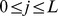. Note that Hamming class 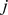 contains genomes carrying 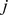 mutations with respect to the master sequence. The frequency of genomes in Hamming class 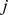 was 
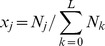
.

#### Shannon entropy

By definition, the per-bit Shannon entropy is 
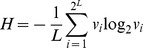
, where 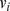 is the frequency of genome *i*. We assumed that transitions alone occurred (see below), thus restricting the total number of distinct genomes to 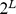 (also see [100]). The Hamming class frequency 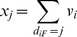, where 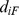 is the Hamming distance of genome 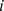 from the master sequence so that the summation extends over all *i* belonging to Hamming class *j*. Because all genomes in a given Hamming class were equally fit we assumed that they were equally likely to occur, so that 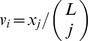, where 
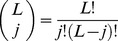
 is the number of possible distinct genomes in Hamming class 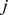. (This assumption neglects the influence of recombination.) Substituting for 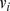 and simplifying yielded 
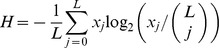
. Note that 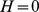 when the master (or any other) sequence alone exists and 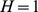 when all possible genomes occur with equal likelihood, the latter signifying an error catastrophe. We evaluated factorials using Stirling's approximation, 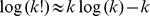 for large values of 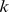 (which sometimes yielded 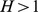).

### Simulation parameters

We employed parameter values representative of HIV-1 infection in vivo [37]. Variations are mentioned below and in the text and figures. We let 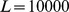 nucleotides and *ρ* = 8.3×10^−4^ crossovers/site/replication [46]. We fixed 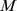 to 3 infections/cell following Jung et al. [24], or let 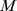 follow a distribution–similar to that observed by Josefsson et al. [69]–where 77% of the cells were singly, 19% doubly, and 4% triply infected [37]. With each 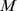, we chose an appropriate 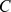 that matched patient data [37]. Following recent estimates of the basic reproductive ratio of HIV-1 *in vivo* [111], we let *P* = 10 infectious progeny virions/cell. A majority of HIV-1 mutations are transitions [70]; as a simplification, we therefore ignored transversions, insertions and deletions. We spanned a wide range of mutation rates in order to identify the error threshold. We let selection follow the fitness landscape derived in [37] to capture corresponding experimental data from [64]. Accordingly, the relative fitness of genome 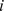 is represented by 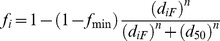, where 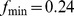 is the minimum fitness of sequences, 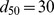 is the Hamming distance at which 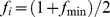, and 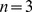 is analogous to the Hill coefficient [37]. The fitness of a virion is determined by the average Hamming distance of its two genomes from the master sequence. We let simulations proceed to 10000 generations (∼30 years).

We examined the influence of variations in some of these parameters as mentioned below.

#### Nucleotide frequencies in HIV-1

In a recent study, 1357 whole genome sequences of HIV-1 were analyzed for their nucleotide composition and found to contain on average ∼36% A's, 24% G's, 18% C's and 22% U's [62]. To mimic this composition, we generated founder sequences by choosing A, G, C and U at each position with probabilities equal to 0.36, 0.24, 0.18 and 0.22, respectively.

#### Nucleotide-specific mutation rates

The frequency of occurrence of different types of mutations in a single round of HIV-1 replication has recently been characterized [63]. In a representative experiment, of the 274 transitions observed, 10.58% were A to G, 53.28% were G to A, 29.56% were C to T and 6.57% were T to C transitions. We implemented nucleotide specific mutation rates mimicking these frequencies as follows. We let 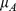, 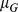, 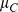, and 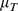 be the mutation rates of A, G, C and T, respectively. (We used T and U interchangeably.) We defined the average mutation rate 
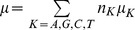
, where 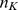 is the frequency of nucleotide *K* in the unmutated sequence. If 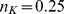 (equal representation of all nucleotides), then 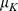 are expected to be proportional to the above frequencies of transitions observed. In other words, 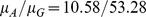, and so on. Using this in the definition of the average mutation rate, we obtained 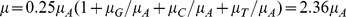, or 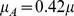. Similarly, we found 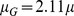, 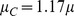, and 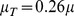. Thus, given 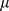, we determined whether a mutation occurred at any position containing a particular nucleotide using the corresponding values of 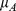, 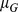, 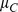, and 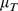. As an approximation, we employed the latter values when 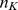 were not all equal as well.

#### Fitness landscape

We performed simulations with two alternative fitness landscapes. First, we modified the fitness landscape above by setting the fitness of genomes below a particular threshold to zero, akin to truncated landscapes employed previously [57], [65], [104], [105]. Thus, 
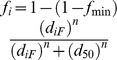
 if 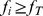 and 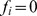 otherwise. We performed simulations with 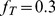 and 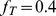 and with 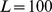 nucleotides.

Second, we followed recent studies of Bonhoeffer and colleagues [66], [67], [102], who assessed the in vitro replicative capacity of about 70000 HIV-1 sequences and argued that the resulting fitness landscape may be described by an equation of the form 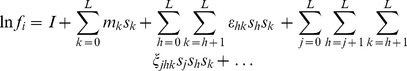, where 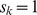 if there is a mutation at position 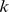 and is zero otherwise; 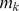 is the loss of fitness due to a mutation at position 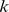; 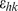 are terms quantifying pair-wise epistatic effects; the third term quantifies ternary effects; and so on. Under conditions when 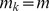, independent of 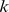, the second term above becomes 
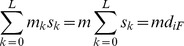
, because the latter summation then simply counts the number of mutations in genome *i*, which is equal to *d_iF_*, the Hamming distance from the master (and also the founder) sequence. Similarly, assuming that pair-wise epistatic effects are also position independent, the third term becomes 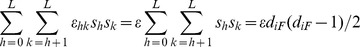, because the latter double summation now counts the number of ways in which two mutations can be chosen from the *d_iF_* mutations in genome *i*. Proceeding similarly, it follows that the above expression for fitness becomes a polynomial in *d_iF_*, namely, 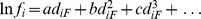 Note that the constant term is set to zero to ensure that the master sequence (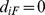) has the maximum relative fitness (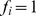). We found that the latter polynomial with terms up to degree 3 provided a good fit to the mean replicative fitness data obtained earlier [64] (Fig. S7). We performed simulations with the resulting best-fit polynomial with non-monotonicities suppressed.

### Predictions of the quasispecies theory

According to the quasispecies theory [7], [8], the structure of the quasispecies is obtained as the dominant eigenvector of the value matrix, 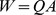. We constructed the mutation matrix 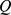 by recognizing that its 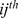 element, 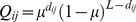, is the probability that genome 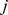 mutates to genome 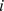, with 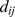 the Hamming distance between genomes 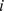 and 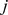. The selection matrix 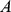 is a diagonal matrix with elements 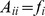, the relative fitness of the respective genomes. We employed three different fitness landscapes: the experimental landscape above, the isolated peak landscape, and the exponential landscape (see above). We computed the dominant eigenvector 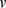 of 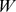 and normalized it so that 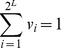. The Hamming class frequencies were then 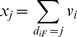. We performed computations using a program written in MATLAB.

## Supporting Information

Figure S1
**Dependence of the structure of the quasispecies on the founder sequence.** Structures of the quasispecies obtained when the founder sequence was the master sequence (circles connected by lines) or was a sequence obtained by mutating the master sequence at 10% of the sites chosen randomly (pluses) with 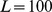 nucleotides for a range of values of 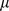 indicated (substitutions/site/replication). The other parameters are the same as in Fig. 1. *Inset* shows the corresponding dependence of the mean steady state Shannon entropy, 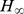, on 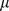 obtained with the master sequence (circles connected by lines) or the mutated sequence (diamonds) as the founder sequence. The structure of the quasispecies and the error threshold are thus not influenced by the choice of the founder sequence.(PDF)

Figure S2
**Quasispecies structure as a function of the genome length.** Structures of the quasispecies obtained with different genome lengths, *L*, and over a range of values of 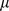 (substitutions/site/replication) indicated. (Some intermediate values of 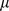 are omitted for clarity.) The corresponding steady state Shannon entropy, 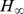, and the resulting dependence of the error threshold, 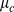, on *L* are presented in Fig. 4.(PDF)

Figure S3
**Quasispecies structure as a function of the population size.** Structures of the quasispecies obtained with different population sizes, *C*, and over a range of values of 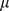 (substitutions/site/replication) indicated. (Some intermediate values of 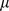 are omitted for clarity.) The corresponding steady state Shannon entropy, 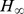, and the resulting dependence of the error threshold, 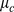, on *C* are presented in Fig. 5.(PDF)

Figure S4
**Quasispecies structure as a function of the recombination rate with M = 3 infections/cell.** Structures of the quasispecies obtained with different recombination rates, 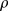 (crossovers/site/replication), and over a range of values of 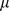 (substitutions/site/replication) indicated. (Some intermediate values of 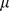 are omitted for clarity.) The corresponding steady state Shannon entropy, 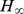, and the resulting dependence of the error threshold, 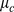, on 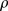 are presented in Figs. 6A and C.(PDF)

Figure S5
**Quasispecies structure as a function of the recombination rate with M∼1 infection/cell.** Structures of the quasispecies obtained with different recombination rates, 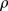 (crossovers/site/replication), and over a range of values of 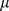 (substitutions/site/replication) indicated, with *M* drawn from a distribution (Methods). (Some intermediate values of 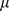 are omitted for clarity.) The corresponding steady state Shannon entropy, 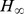, and the resulting dependence of the error threshold, 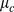, on 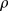 are presented in Figs. 6B and D.(PDF)

Figure S6
**Dependence of the error threshold on the nucleotide composition of the founder sequence and nucleotide-specific mutation rates.** The mean steady state Shannon entropy, 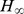, as a function of the mutation rate 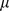 obtained with the founder sequence containing all nucleotides with equal frequencies and mutating at equal rates (blue), reproduced from Fig. 2C (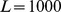 nucleotides). The corresponding 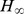 when the founder sequence contained nucleotides at frequencies representative of HIV-1 (∼36% A's, 24% G's, 18% C's and 22% U's) mutating at equal rates (green) or at nucleotide-specific rates (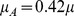, 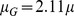, 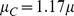, and 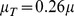) (red). The other parameters are the same as in Fig. 2C.(PDF)

Figure S7
**Dependence of the error threshold on the fitness landscape.** (A) The mean steady state Shannon entropy, 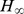, as a function of the mutation rate 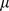 obtained with the fitness landscape 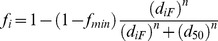 if 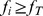 and 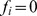 otherwise, with 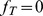 (red), 0.3 (blue), and 0.4 (green). Note that 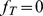 corresponds to the simulations in Fig. S1. With larger lengths, the fitness landscape has to be appropriately rescaled to avoid the extinction of the viral population due to severe fitness penalties (not shown). (B) 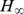 as a function of 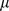 obtained with the landscape 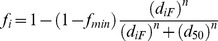 (blue) and the polynomial fitness landscape 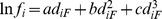 (red). Note that the former data is the same as in Fig. 2C with 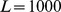 nucleotides. *Inset* in (B) shows best fits of the two landscapes (blue and red lines, respectively) to data (symbols) excluding outliers (open symbols) from Bonhoeffer et al. (Science 306: 1547–1550 (2004)) modified to account for the observed frequencies of synonymous and non-synonymous mutations (see Balagam et al., PLoS ONE 6: e14531 (2011)). The best-fit parameter estimates are 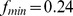, 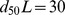 and 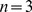 (blue); and 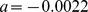, 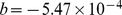 and 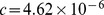 (red). Because data was available only until Hamming distance ∼90 to which the polynomial can be fit, extrapolating the polynomial to higher Hamming distances yielded an unrealistic increase of fitness. To avoid this non-monotonic behavior, the fitness of genomes beyond the minimum (which occurred at Hamming distance 82) was set equal to the minimum.(PDF)

Figure S8
**Quasispecies structures yielding estimates of the error threshold of HIV-1 with M = 3 infections/cell.** Structures of the quasispecies obtained with different population sizes, *C*, and over a range of values of 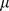 (substitutions/site/replication) indicated. (Some intermediate values of 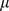 are omitted for clarity.) The corresponding dependence of the steady state Shannon entropy, 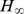, on 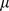 is presented in Fig. 8A.(PDF)

Figure S9
**Quasispecies structures yielding estimates of the error threshold of HIV-1 with M∼1 infection/cell.** Structures of the quasispecies obtained with different population sizes, *C*, and over a range of values of 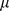 (substitutions/site/replication) indicated, with *M* drawn from a distribution (Methods). (Some intermediate values of 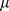 are omitted for clarity.) The corresponding dependence of the steady state Shannon entropy, 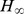, on 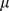 is presented in Fig. 8B.(PDF)

Text S1 **The computer program employed for our simulations.**
(PDF)

## References

[1] Coffin JM (1995) HIV population-dynamics in-vivo: Implications for genetic-variation, pathogenesis, and therapy. Science 267: 483–489. 7824947 10.1126/science.7824947

[2] Rambaut A, Posada D, Crandall KA, Holmes EC (2004) The causes and consequences of HIV evolution. Nat Rev Genet 5: 52–61. 14708016 10.1038/nrg1246

[3] Mehellou Y, De Clercq E (2009) Twenty-six years of anti-HIV drug discovery: Where do we stand and where do we go? J Med Chem 53: 521–538. 10.1021/jm900492g 19785437

[4] Berkhout B, Sanders RW (2011) Molecular strategies to design an escape-proof antiviral therapy. Antivir Res 92: 7–14. 21513746 10.1016/j.antiviral.2011.04.002

[5] Walker BD, Burton DR (2008) Toward an AIDS vaccine. Science 320: 760–764. 18467582 10.1126/science.1152622

[6] Korber B (2011) Building on the past to define an efficient path to an HIV vaccine. Expert Rev Vaccines 10: 929–931. 21806390 10.1586/erv.11.81

[7] Eigen M (1971) Selforganization of matter and the evolution of biological macromolecules. Die Naturwissenschaften 58: 456–523. 10.1007/BF00623322 4942363

[8] Eigen M, McCaskill J, Schuster P (1989) The molecular quasi-species. Adv Chem Phys 75: 149–263.

[9] Crotty S, Cameron CE, Andino R (2001) RNA virus error catastrophe: Direct molecular test by using ribavirin. Proc Natl Acad Sci U S A 98: 6895–6900. 11371613 10.1073/pnas.111085598 PMC34449

[10] Sierra S, Davila M, Lowenstein PR, Domingo E (2000) Response of foot-and-mouth disease virus to increased mutagenesis: Influence of viral load and fitness in loss of infectivity. J Virol 74: 8316–8323. 10954530 10.1128/jvi.74.18.8316-8323.2000 PMC116341

[11] Grande-Perez A, Sierra S, Castro MG, Domingo E, Lowenstein PR (2002) Molecular indetermination in the transition to error catastrophe: Systematic elimination of lymphocytic choriomeningitis virus through mutagenesis does not correlate linearly with large increases in mutant spectrum complexity. Proc Natl Acad Sci U S A 99: 12938–12943. 12215495 10.1073/pnas.182426999 PMC130564

[12] Grande-Perez A, Gomez-Mariano G, Lowenstein PR, Domingo E (2005) Mutagenesis-induced, large fitness variations with an invariant arenavirus consensus genomic nucleotide sequence. J Virol 79: 10451–10459. 16051837 10.1128/JVI.79.16.10451-10459.2005 PMC1182645

[13] Grande-Perez A, Lazaro E, Lowenstein P, Domingo E, Manrubia SC (2005) Suppression of viral infectivity through lethal defection. Proc Natl Acad Sci U S A 102: 4448–4452. 15767582 10.1073/pnas.0408871102 PMC555496

[14] Loeb LA, Essigmann JM, Kazazi F, Zhang J, Rose KD, et al (1999) Lethal mutagenesis of HIV with mutagenic nucleoside analogs. Proc Natl Acad Sci U S A 96: 1492–1497. 9990051 10.1073/pnas.96.4.1492 PMC15492

[15] Harris KS, Brabant W, Styrchak S, Gall A, Daifuku R (2005) KP-1212/1461, a nucleoside designed for the treatment of HIV by viral mutagenesis. Antivir Res 67: 1–9. 15890415 10.1016/j.antiviral.2005.03.004

[16] Smith RA, Loeb LA, Preston BD (2005) Lethal mutagenesis of HIV. Virus Res 107: 215–228. 15649567 10.1016/j.virusres.2004.11.011

[17] Dapp MJ, Clouser CL, Patterson S, Mansky LM (2009) 5-Azacytidine can induce human immunodeficiency virus type 1 lethal mutagenesis. J Virol 83: 11950–11958. 19726509 10.1128/JVI.01406-09 PMC2772699

[18] Mullins JI, Heath L, Hughes JP, Kicha J, Styrchak S, et al (2011) Mutation of HIV-1 genomes in a clinical population treated with the mutagenic nucleoside KP1461. PLoS ONE 6: e15135. 21264288 10.1371/journal.pone.0015135 PMC3021505

[19] Harris RS, Liddament MT (2004) Retroviral restriction by APOBEC proteins. Nat Rev Immunol 4: 868–877. 15516966 10.1038/nri1489

[20] Malim MH (2009) APOBEC proteins and intrinsic resistance to HIV-1 infection. Philos T Roy Soc B 364: 675–687. 10.1098/rstb.2008.0185 PMC2660912 19038776

[21] Nathans R, Cao H, Sharova N, Ali A, Sharkey M, et al (2008) Small-molecule inhibition of HIV-1 Vif. Nat Biotech 26: 1187–1192. 10.1038/nbt.1496 PMC2693000 18806783

[22] Pillai S, Wong J, Barbour J (2008) Turning up the volume on mutational pressure: Is more of a good thing always better? (A case study of HIV-1 Vif and APOBEC3). Retrovirology 5: 26. 18339206 10.1186/1742-4690-5-26 PMC2323022

[23] Suspene R, Sommer P, Henry M, Ferris S, Guetard D, et al (2004) APOBEC3G is a single-stranded DNA cytidine deaminase and functions independently of HIV reverse transcriptase. Nucleic Acids Res 32: 2421–2429. 15121899 10.1093/nar/gkh554 PMC419444

[24] Jung A, Maier R, Vartanian JP, Bocharov G, Jung V, et al (2002) Multiply infected spleen cells in HIV patients. Nature 418: 144–144. 12110879 10.1038/418144a

[25] Levy DN, Aldrovandi GM, Kutsch O, Shaw GM (2004) Dynamics of HIV-1 recombination in its natural target cells. Proc Natl Acad Sci U S A 101: 4204–4209. 15010526 10.1073/pnas.0306764101 PMC384719

[26] McCutchan FE (2006) Global epidemiology of HIV. J Med Virol 78: S7–S12. 16622870 10.1002/jmv.20599

[27] Onafuwa-Nuga A, Telesnitsky A (2009) The remarkable frequency of human immunodeficiency virus type 1 genetic recombination. Microbiol Mol Biol Rev 73: 451–480. 19721086 10.1128/MMBR.00012-09 PMC2738136

[28] Brown AJL (1997) Analysis of HIV-1 env gene sequences reveals evidence for a low effective number in the viral population. Proc Natl Acad Sci U S A 94: 1862–1865. 9050870 10.1073/pnas.94.5.1862 PMC20008

[29] Nijhuis M, Boucher CAB, Schipper P, Leitner T, Schuurman R, et al (1998) Stochastic processes strongly influence HIV-1 evolution during suboptimal protease-inhibitor therapy. Proc Natl Acad Sci U S A 95: 14441–14446. 9826719 10.1073/pnas.95.24.14441 PMC24392

[30] Rodrigo AG, Shpaer EG, Delwart EL, Iversen AKN, Gallo MV, et al (1999) Coalescent estimates of HIV-1 generation time in vivo. Proc Natl Acad Sci U S A 96: 2187–2191. 10051616 10.1073/pnas.96.5.2187 PMC26758

[31] Rouzine IM, Coffin JM (1999) Linkage disequilibrium test implies a large effective population number for HIV in vivo. Proc Natl Acad Sci U S A 96: 10758–10763. 10485899 10.1073/pnas.96.19.10758 PMC17956

[32] Seo TK, Thorne JL, Hasegawa M, Kishino H (2002) Estimation of effective population size of HIV-1 within a host: A pseudomaximum-likelihood approach. Genetics 160: 1283–1293. 11973287 10.1093/genetics/160.4.1283 PMC1462041

[33] Achaz G, Palmer S, Kearney M, Maldarelli F, Mellors JW, et al (2004) A robust measure of HIV-1 population turnover within chronically infected individuals. Mol Biol Evol 21: 1902–1912. 15215321 10.1093/molbev/msh196

[34] Shriner D, Shankarappa R, Jensen MA, Nickle DC, Mittler JE, et al (2004) Influence of random genetic drift on human immunodeficiency virus type I env evolution during chronic infection. Genetics 166: 1155–1164. 15082537 10.1534/genetics.166.3.1155 PMC1470792

[35] Kouyos RD, Althaus CL, Bonhoeffer S (2006) Stochastic or deterministic: what is the effective population size of HIV-1? Trends Microbiol 14: 507–511. 17049239 10.1016/j.tim.2006.10.001

[36] Liu Y, Mittler J (2008) Selection dramatically reduces effective population size in HIV-1 infection. BMC Evol Biol 8: 133. 18454872 10.1186/1471-2148-8-133 PMC2396635

[37] Balagam R, Singh V, Sagi AR, Dixit NM (2011) Taking multiple infections of cells and recombination into account leads to small within-host effective-population-size estimates of HIV-1. PLoS ONE 6: e14531. 21249189 10.1371/journal.pone.0014531 PMC3020941

[38] Boerlijst MC, Bonhoeffer S, Nowak MA (1996) Viral quasi-species and recombination. P Roy Soc Lond B Bio 263: 1577–1584.

[39] Bretscher MT, Althaus CL, Muller V, Bonhoeffer S (2004) Recombination in HIV and the evolution of drug resistance: for better or for worse? BioEssays 26: 180–188. 14745836 10.1002/bies.10386

[40] Althaus CL, Bonhoeffer S (2005) Stochastic interplay between mutation and recombination during the acquisition of drug resistance mutations in human immunodeficiency virus type 1. J Virol 79: 13572–13578. 16227277 10.1128/JVI.79.21.13572-13578.2005 PMC1262575

[41] Bocharov G, Ford NJ, Edwards J, Breinig T, Wain-Hobson S, et al (2005) A genetic-algorithm approach to simulating human immunodeficiency virus evolution reveals the strong impact of multiply infected cells and recombination. J Gen Virol 86: 3109–3118. 16227234 10.1099/vir.0.81138-0

[42] Fraser C (2005) HIV recombination: what is the impact on antiretroviral therapy? J Roy Soc Interface 2: 489–503. 16849208 10.1098/rsif.2005.0064 PMC1618498

[43] Rouzine IM, Coffin JM (2005) Evolution of human immunodeficiency virus under selection and weak recombination. Genetics 170: 7–18. 15744057 10.1534/genetics.104.029926 PMC1449738

[44] Carvajal-Rodriguez A, Crandall KA, Posada D (2007) Recombination favors the evolution of drug resistance in HIV-1 during antiretroviral therapy. Infect Genet Evol 7: 476–483. 17369105 10.1016/j.meegid.2007.02.001 PMC2041866

[45] Gheorghiu-Svirschevski S, Rouzine IM, Coffin JM (2007) Increasing sequence correlation limits the efficiency of recombination in a multisite evolution model. Mol Biol Evol 24: 574–586. 17138627 10.1093/molbev/msl189

[46] Suryavanshi GW, Dixit NM (2007) Emergence of recombinant forms of HIV: Dynamics and scaling. PLoS Comput Biol 3: e205. 10.1371/journal.pcbi.0030205 PMC2041978 17967052

[47] Vijay NNV, Vasantika, Ajmani R, Perelson AS, Dixit NM (2008) Recombination increases human immunodeficiency virus fitness, but not necessarily diversity. J Gen Virol 89: 1467–1477. 18474563 10.1099/vir.0.83668-0

[48] Arora P, Dixit NM (2009) Timing the emergence of resistance to anti-HIV drugs with large genetic barriers. PLoS Comput Biol 5: e1000305. 19282958 10.1371/journal.pcbi.1000305 PMC2643484

[49] Gadhamsetty S, Dixit NM (2010) Estimating frequencies of minority nevirapine-resistant strains in chronically HIV-1-infected individuals naive to nevirapine by using stochastic simulations and a mathematical model. J Virol 84: 10230–10240. 20668070 10.1128/JVI.01010-10 PMC2937761

[50] Rouzine IM, Coffin JM (2010) Multi-site adaptation in the presence of infrequent recombination. Theor Popul Biol 77: 189–204. 20149814 10.1016/j.tpb.2010.02.001 PMC2849900

[51] Batorsky R, Kearney MF, Palmer SE, Maldarelli F, Rouzine IM, et al (2011) Estimate of effective recombination rate and average selection coefficient for HIV in chronic infection. Proc Natl Acad Sci U S A 108: 5661–5666. 21436045 10.1073/pnas.1102036108 PMC3078368

[52] Lee HY, Giorgi EE, Keele BF, Gaschen B, Athreya GS, et al (2009) Modeling sequence evolution in acute HIV-1 infection. J Theor Biol 261: 341–360. 19660475 10.1016/j.jtbi.2009.07.038 PMC2760689

[53] Pearson JE, Krapivsky P, Perelson AS (2011) Stochastic theory of early viral infection: Continuous versus burst production of virions. PLoS Comput Biol 7: e1001058. 21304934 10.1371/journal.pcbi.1001058 PMC3033366

[54] Wagner GP, Krall P (1993) What is the difference between models of error thresholds and Muller's ratchet? J Math Biol 32: 33–44.

[55] Woodcock G, Higgs PG (1996) Population evolution on a multiplicative single-peak fitness landscape. J Theor Biol 179: 61–73. 8733432 10.1006/jtbi.1996.0049

[56] Wilke C (2005) Quasispecies theory in the context of population genetics. BMC Evol Biol 5: 44. 16107214 10.1186/1471-2148-5-44 PMC1208876

[57] Summers J, Litwin S (2006) Examining the theory of error catastrophe. J Virol 80: 20–26. 16352527 10.1128/JVI.80.1.20-26.2006 PMC1317512

[58] Takeuchi N, Hogeweg P (2007) Error-threshold exists in fitness landscapes with lethal mutants. BMC Evol Biol 7: 15. 17286853 10.1186/1471-2148-7-15 PMC1805495

[59] Nowak M, Schuster P (1989) Error thresholds of replication in finite populations-Mutation frequencies and the onset of Muller's ratchet. J Theor Biol 137: 375–395. 2626057 10.1016/s0022-5193(89)80036-0

[60] Bonhoeffer S, Stadler PF (1993) Error thresholds on correlated fitness landscapes. J Theor Biol 164: 359–372.

[61] Neher RA, Leitner T (2010) Recombination rate and selection strength in HIV intra-patient evolution. PLoS Comput Biol 6: e1000660. 20126527 10.1371/journal.pcbi.1000660 PMC2813257

[62] Pandit A, Sinha S (2011) Differential trends in the codon usage patterns in HIV-1 genes. PLoS ONE 6: e28889. 22216135 10.1371/journal.pone.0028889 PMC3245234

[63] Abram ME, Ferris AL, Shao W, Alvord WG, Hughes SH (2010) Nature, position, and frequency of mutations made in a single cycle of HIV-1 replication. J Virol 84: 9864–9878. 20660205 10.1128/JVI.00915-10 PMC2937799

[64] Bonhoeffer S, Chappey C, Parkin NT, Whitcomb JM, Petropoulos CJ (2004) Evidence for positive epistasis in HIV-1. Science 306: 1547–1550. 15567861 10.1126/science.1101786

[65] Saakian DB, Biebricher CK, Hu C-K (2009) Phase diagram for the Eigen quasispecies theory with a truncated fitness landscape. Phys Rev E 79: 041905. 10.1103/PhysRevE.79.041905 19518254

[66] Hinkley T, Martins J, Chappey C, Haddad M, Stawiski E, et al (2011) A systems analysis of mutational effects in HIV-1 protease and reverse transcriptase. Nat Genet 43: 487–489. 21441930 10.1038/ng.795

[67] Kouyos RD, Leventhal GE, Hinkley T, Haddad M, Whitcomb JM, et al (2012) Exploring the complexity of the HIV-1 fitness landscape. PLoS Genet 8: e1002551. 22412384 10.1371/journal.pgen.1002551 PMC3297571

[68] Shankarappa R, Margolick JB, Gange SJ, Rodrigo AG, Upchurch D, et al (1999) Consistent viral evolutionary changes associated with the progression of human immunodeficiency virus type 1 infection. J Virol 73: 10489–10502. 10559367 10.1128/jvi.73.12.10489-10502.1999 PMC113104

[69] Josefsson L, King MS, Makitalo B, Brannstrom J, Shao W, et al (2011) Majority of CD4+ T cells from peripheral blood of HIV-1-infected individuals contain only one HIV DNA molecule. Proc Natl Acad Sci U S A 108: 11199–11204. 21690402 10.1073/pnas.1107729108 PMC3131354

[70] Mansky LM, Temin HM (1995) Lower in-vivo mutation-rate of human-immunodeficiency-virus type-1 than that predicted from the fidelity of purified reverse-transcriptase. J Virol 69: 5087–5094. 7541846 10.1128/jvi.69.8.5087-5094.1995 PMC189326

[71] Domingo E, Biebricher CK, Eigen M, Holland JJ (2001) Quasispecies and RNA virus evolution: Principles and consequences: Georgetown, TX: Landes Bioscience.

[72] Wilke CO, Wang JL, Ofria C, Lenski RE, Adami C (2001) Evolution of digital organisms at high mutation rate leads to survival of the flattest. Nature 412: 331–333. 11460163 10.1038/35085569

[73] Vignuzzi M, Wendt E, Andino R (2008) Engineering attenuated virus vaccines by controlling replication fidelity. Nat Med 14: 154–161. 18246077 10.1038/nm1726

[74] Lauring AS, Andino R (2010) Quasispecies theory and the behavior of RNA viruses. PLoS Pathog 6: e1001005. 20661479 10.1371/journal.ppat.1001005 PMC2908548

[75] Eigen M (2002) Error catastrophe and antiviral strategy. Proc Natl Acad Sci U S A 99: 13374–13376. 12370416 10.1073/pnas.212514799 PMC129678

[76] Wiehe T (1997) Model dependency of error thresholds: the role of fitness functions and contrasts between the finite and infinite sites models. Genet Res 69: 127–136.

[77] van Nimwegen E, Crutchfield JP, Mitchell M (1999) Statistical dynamics of the royal road genetic algorithm. Theor Comput Sci 229: 41–102.

[78] Saakian DB, Hu C-K (2006) Exact solution of the Eigen model with general fitness functions and degradation rates. Proc Natl Acad Sci USA 103: 4935–4939. 16549804 10.1073/pnas.0504924103 PMC1458773

[79] Bonnaz D, Koch AJ (1998) Stochastic model of evolving populations. J Phys A- Math Gen 31: 417–429.

[80] Alves D, Fontanari JF (1998) Error threshold in finite populations. Phys Rev E 57: 7008–7013.

[81] Campos PRA, Fontanari JF (1998) Finite-size scaling of the quasispecies model. Phys Rev E 58: 2664–2667.

[82] Campos PRA, Fontanari JF (1999) Finite-size scaling of the error threshold transition in finite populations. J Phys A 32: L1–L7.

[83] Ray TS, Payne KA, Moseley LL (2008) Role of finite populations in determining evolutionary dynamics. Phys Rev E 77: 021909. 10.1103/PhysRevE.77.021909 18352053

[84] Park J-M, Munoz E, Deem MW (2010) Quasispecies theory for finite populations. Phys Rev E 81: 011902. 10.1103/PhysRevE.81.011902 PMC4479305 20365394

[85] Saakian DB, Deem MW, Hu C-K (2012) Finite population size effects in quasispecies models with single-peak fitness landscape. Europhy Lett 98: 18001.

[86] Dixit NM, Srivastava P, Vishnoi NK (2012) A finite population model of molecular evolution: Theory and computation. J Comput Biol In press. 10.1089/cmb.2012.0064 23057826

[87] Dixit NM (2008) Modelling HIV infection dynamics: The role of recombination in the development of drug resistance. Future HIV Therapy 2: 375–388.

[88] Barton NH, Charlesworth B (1998) Why sex and recombination? Science 281: 1986–1990. 9748151

[89] Otto SP, Lenormand T (2002) Resolving the paradox of sex and recombination. Nat Rev Genet 3: 252–261. 11967550 10.1038/nrg761

[90] Keightley PD, Otto SP (2006) Interference among deleterious mutations favours sex and recombination in finite populations. Nature 443: 89–92. 16957730 10.1038/nature05049

[91] Hill WG, Robertson A (1966) Effect of linkage on limits to artificial selection. Genet Res 8: 269–294. 5980116

[92] Ewens WJ (2004) Mathematical population genetics. New York: Springer.

[93] Hartl DL, Clark AG (2007) Principles of population genetics. Sunderland, MA: Sinauer Associates, Inc.

[94] Kouyos RD, Silander OK, Bonhoeffer S (2007) Epistasis between deleterious mutations and the evolution of recombination. Trends Ecol Evol 22: 308–315. 17337087 10.1016/j.tree.2007.02.014

[95] Haase AT, Henry K, Zupancic M, Sedgewick G, Faust RA, et al (1996) Quantitative image analysis of HIV-1 infection in lymphoid tissue. Science 274: 985–989. 8875941 10.1126/science.274.5289.985

[96] Charlesworth B (2009) Effective population size and patterns of molecular evolution and variation. Nat Rev Genet 10: 195–205. 19204717 10.1038/nrg2526

[97] Voronin Y, Holte S, Overbaugh J, Emerman M (2009) Genetic drift of HIV populations in culture. PLoS Genet 5: e1000431. 19300501 10.1371/journal.pgen.1000431 PMC2652835

[98] Gillespie JH (2000) Genetic drift in an infinite population: The pseudohitchhiking model. Genetics 155: 909–919. 10835409 10.1093/genetics/155.2.909 PMC1461093

[99] Frost SDW, Dumaurier MJ, Wain-Hobson S, Brown AJL (2001) Genetic drift and within-host metapopulation dynamics of HIV-1 infection. Proc Natl Acad Sci USA 98: 6975–6980. 11381143 10.1073/pnas.131056998 PMC34518

[100] Swetina J, Schuster P (1982) Self-replication with errors: a model for polynucleotide replication. Biophys Chem 16: 329–345. 7159681 10.1016/0301-4622(82)87037-3

[101] Dahirel V, Shekhar K, Pereyra F, Miura T, Artyomov M, et al (2011) Coordinate linkage of HIV evolution reveals regions of immunological vulnerability. Proc Natl Acad Sci USA 108: 11530–11535. 21690407 10.1073/pnas.1105315108 PMC3136285

[102] Kouyos RD, von Wyl V, Hinkley T, Petropoulos CJ, Haddad M, et al (2011) Assessing predicted HIV-1 replicative capacity in a clinical setting. PLoS Pathog 7: e1002321. 22072960 10.1371/journal.ppat.1002321 PMC3207887

[103] Sanjuan R, Moya A, Elena SF (2004) The distribution of fitness effects caused by single-nucleotide substitutions in an RNA virus. Proc Natl Acad Sci USA 101: 8396–8401. 15159545 10.1073/pnas.0400146101 PMC420405

[104] Kirakosyan Z, Saakian DB, Hu C-K (2010) Evolution models with lethal mutations on symmetric or random fitness landscapes. Phys Rev E 82: 011904. 10.1103/PhysRevE.82.011904 20866645

[105] Saakian DB, Biebricher CK, Hu C-K (2011) Lethal mutants and truncated selection together solve a paradox of the origin of life. PLoS ONE 6: e21904. 21814563 10.1371/journal.pone.0021904 PMC3144202

[106] Fan J, Negroni M, Robertson DL (2007) The distribution of HIV-1 recombination breakpoints. Infect Genet Evol 7: 717–723. 17851137 10.1016/j.meegid.2007.07.012

[107] Archer J, Pinney JW, Fan J, Simon-Loriere E, Arts EJ, et al (2008) Identifying the important HIV-1 recombination breakpoints. PLoS Comput Biol 4: e1000178. 18787691 10.1371/journal.pcbi.1000178 PMC2522274

[108] Bull JJ, Sanjuan R, Wilke CO (2007) Theory of lethal mutagenesis for viruses. J Virol 81: 2930–2939. 17202214 10.1128/JVI.01624-06 PMC1865999

[109] Zeldovich KB, Chen P, Shakhnovich EI (2007) Protein stability imposes limits on organism complexity and speed of molecular evolution. Proc Natl Acad Sci U S A 104: 16152–16157. 17913881 10.1073/pnas.0705366104 PMC2042177

[110] Chen P, Shakhnovich EI (2009) Lethal mutagenesis in viruses and bacteria. Genetics 183: 639–650. 19620390 10.1534/genetics.109.106492 PMC2766323

[111] Ribeiro RM, Qin L, Chavez LL, Li D, Self SG, et al (2010) Estimation of the initial viral growth rate and basic reproductive number during acute HIV-1 infection. J Virol 84: 6096–6102. 20357090 10.1128/JVI.00127-10 PMC2876646

