# Supplementary figures and images for: Stochastic Simulations Suggest that HIV-1 Survives Close to Its Error Threshold
Source: PLoS Comput Biol. 2012 Sep 13;8(9):e1002684. doi: 10.1371/journal.pcbi.1002684 (PMC3441496; doi:10.1371/journal.pcbi.1002684)

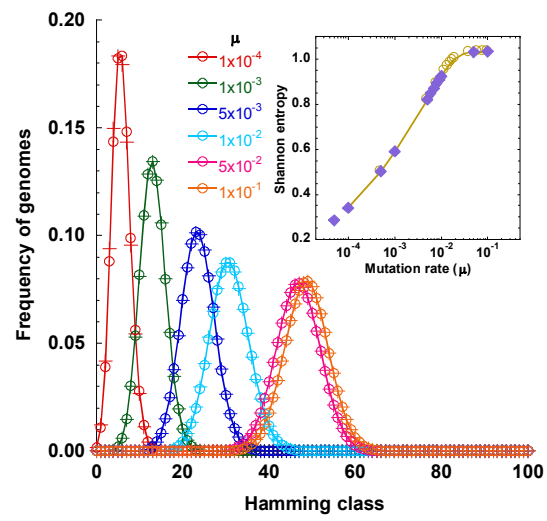

Supplement: Figure S1 — Dependence of the structure of the quasispecies on the founder sequence. Structures of the quasispecies obtained when the founder sequence was the master sequence (circles connected by lines) or was a sequence obtained by mutating the master sequence at 10% of the sites chosen randomly (pluses) with nucleotides for a range of values of indicated (substitutions/site/replication). The other parameters are the same as in Fig. 1. Inset shows the corresponding dependence of the mean steady state Shannon entropy, , on obtained with the master sequence (circles connected by lines) or the mutated sequence (diamonds) as the founder sequence. The structure of the quasispecies and the error threshold are thus not influenced by the choice of the founder sequence. (PDF) [file pcbi.1002684.s001.pdf]

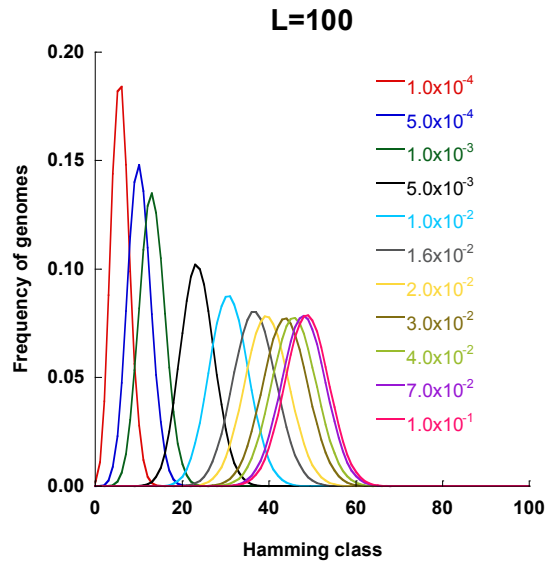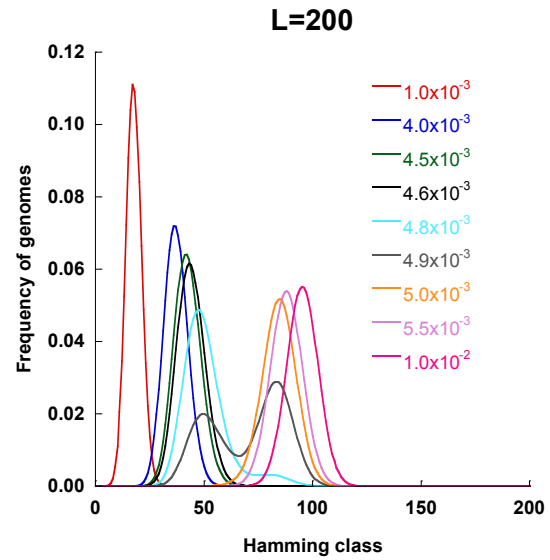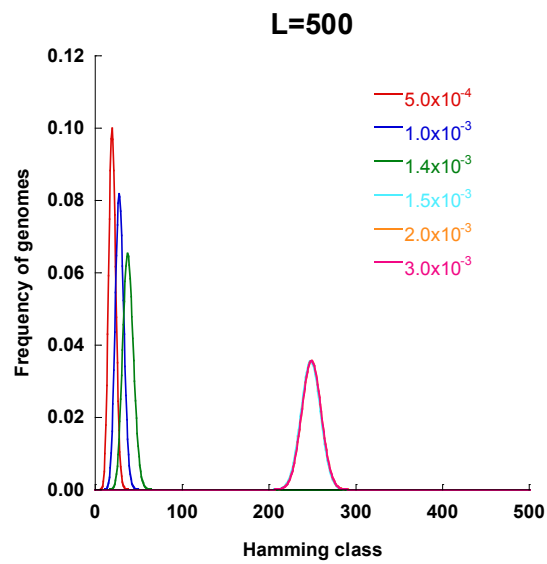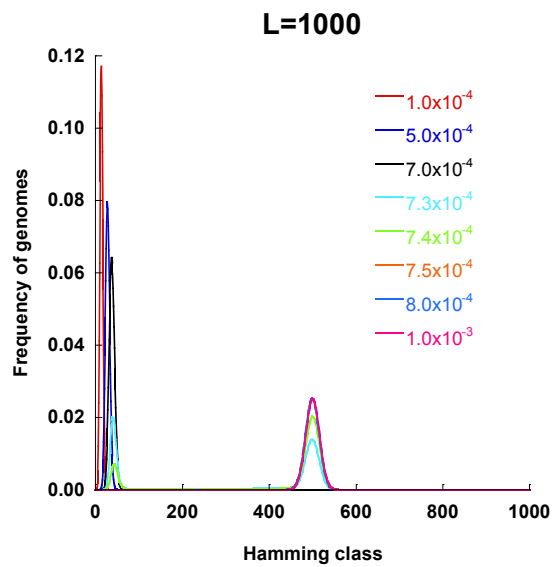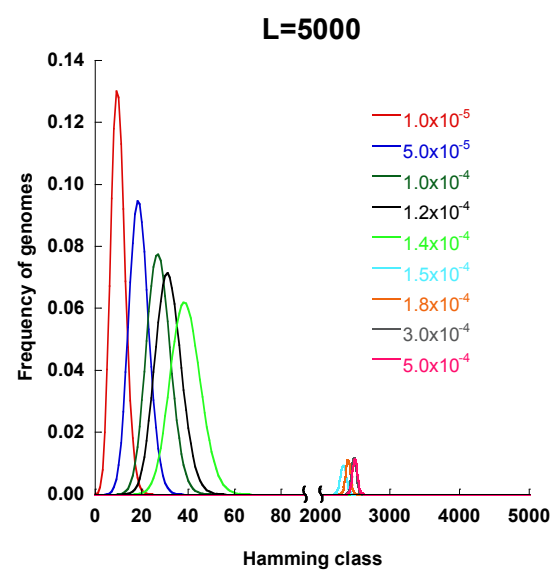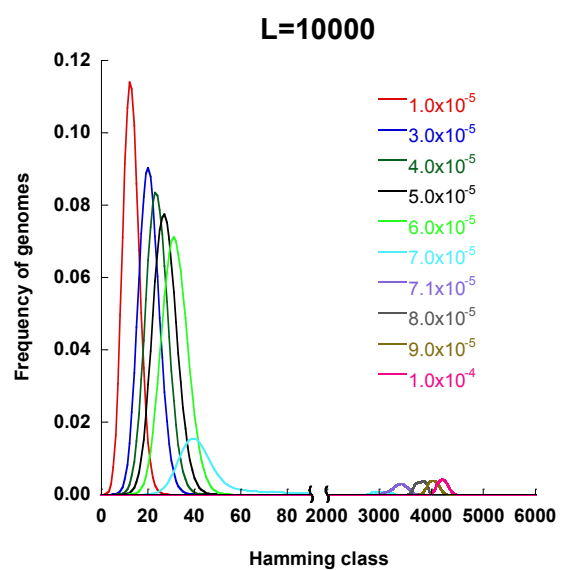

Supplement: Figure S2 — Quasispecies structure as a function of the genome length. Structures of the quasispecies obtained with different genome lengths, L, and over a range of values of (substitutions/site/replication) indicated. (Some intermediate values of are omitted for clarity.) The corresponding steady state Shannon entropy, , and the resulting dependence of the error threshold, , on L are presented in Fig. 4. (PDF) [file pcbi.1002684.s002.pdf]

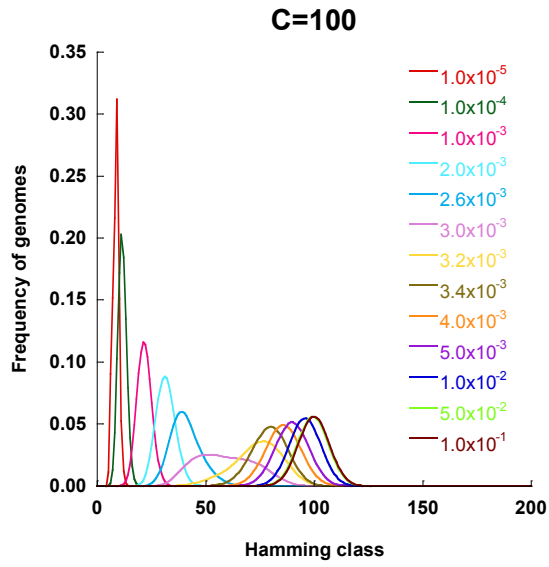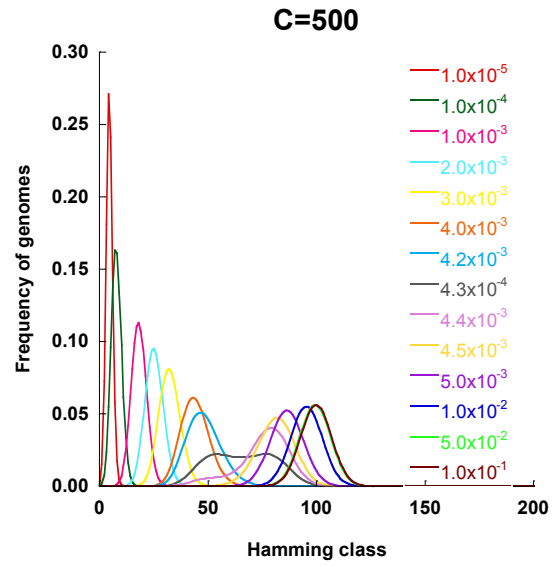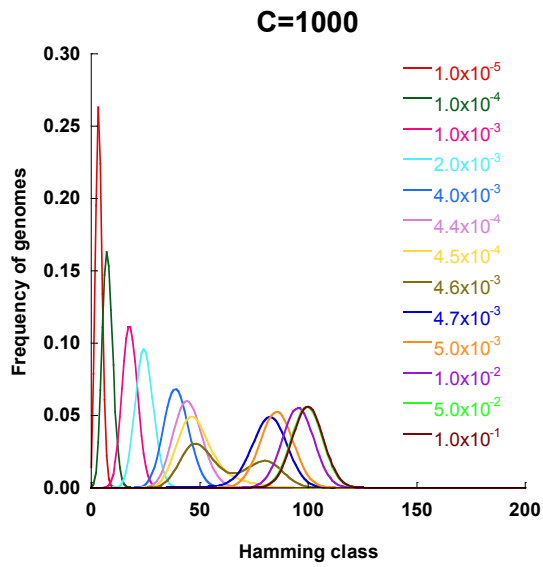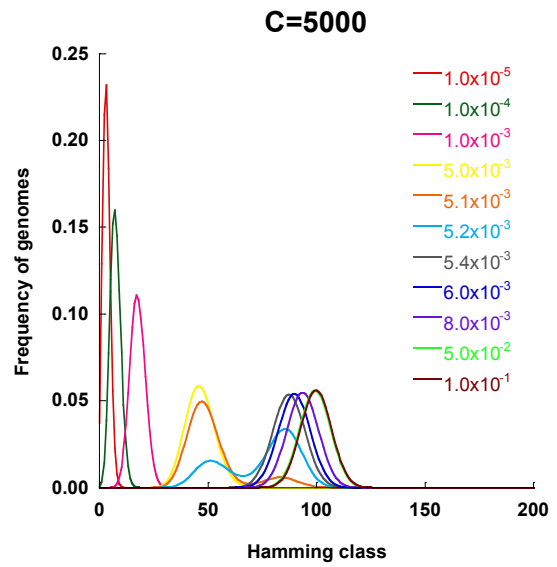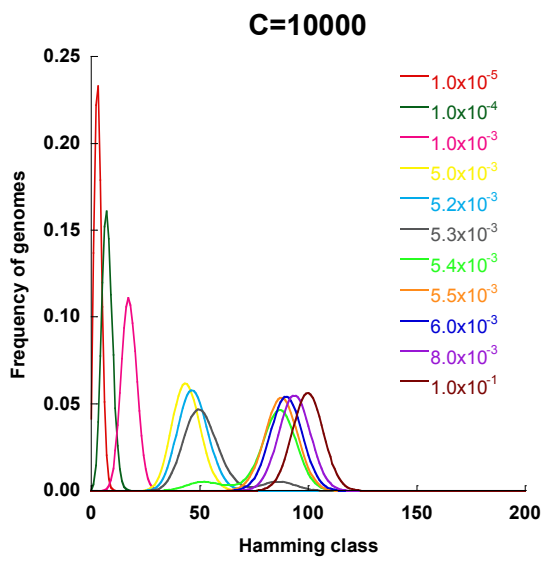

Supplement: Figure S3 — Quasispecies structure as a function of the population size. Structures of the quasispecies obtained with different population sizes, C, and over a range of values of (substitutions/site/replication) indicated. (Some intermediate values of are omitted for clarity.) The corresponding steady state Shannon entropy, , and the resulting dependence of the error threshold, , on C are presented in Fig. 5. (PDF) [file pcbi.1002684.s003.pdf]

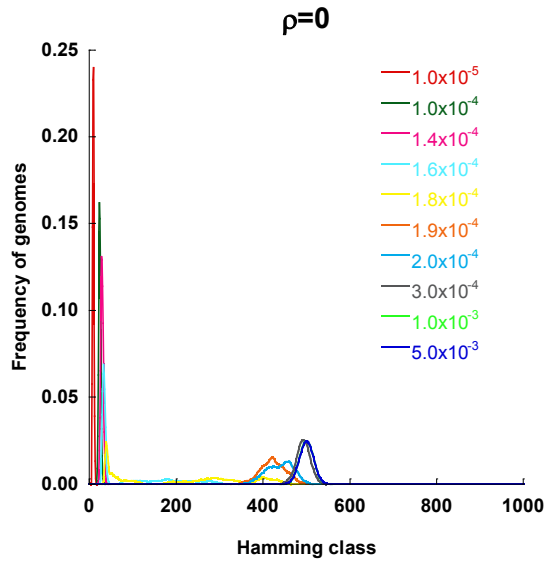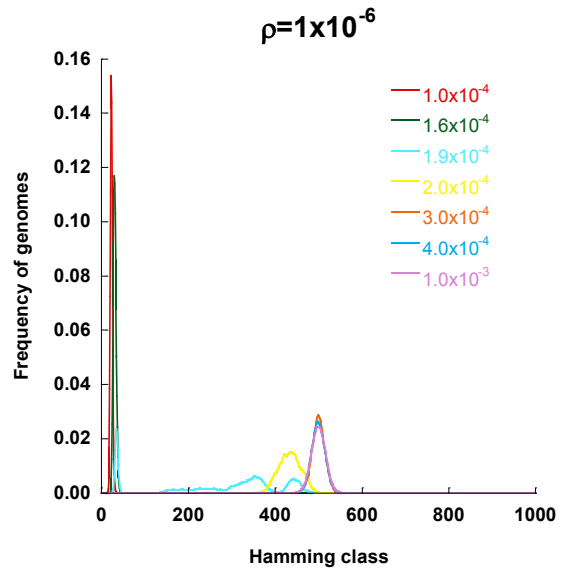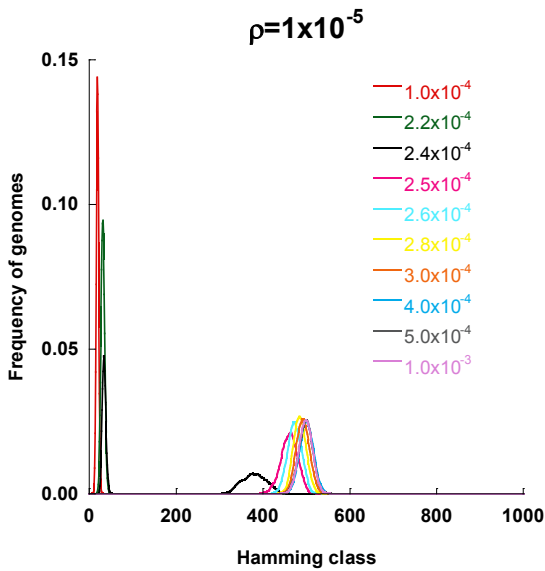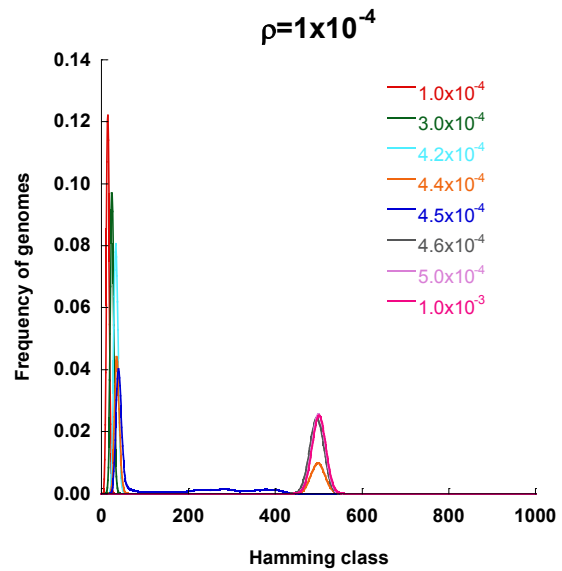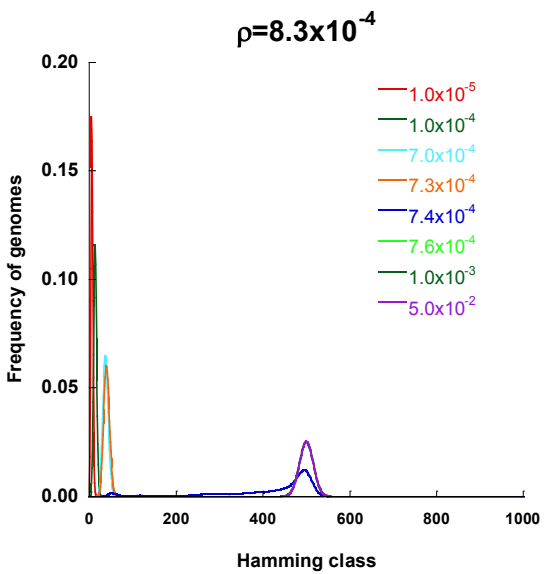

Supplement: Figure S4 — Quasispecies structure as a function of the recombination rate with M = 3 infections/cell. Structures of the quasispecies obtained with different recombination rates, (crossovers/site/replication), and over a range of values of (substitutions/site/replication) indicated. (Some intermediate values of are omitted for clarity.) The corresponding steady state Shannon entropy, , and the resulting dependence of the error threshold, , on are presented in Figs. 6A and C. (PDF) [file pcbi.1002684.s004.pdf]

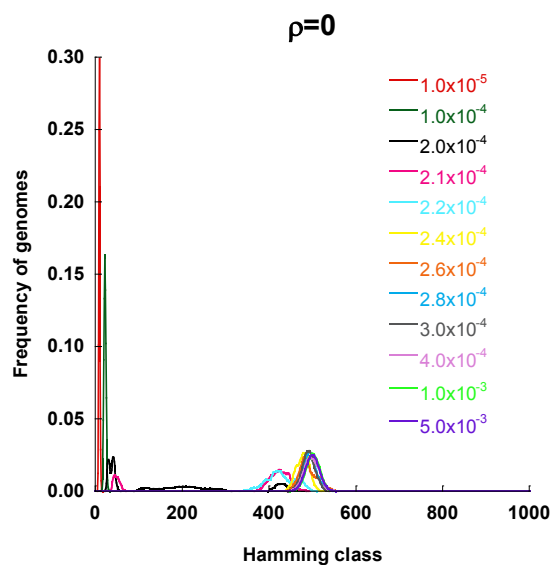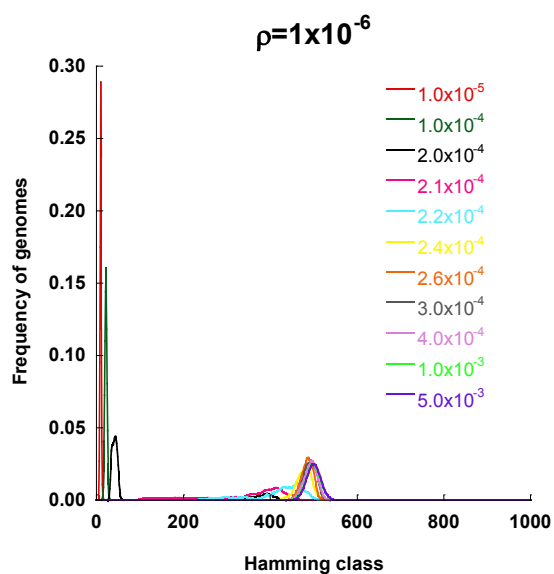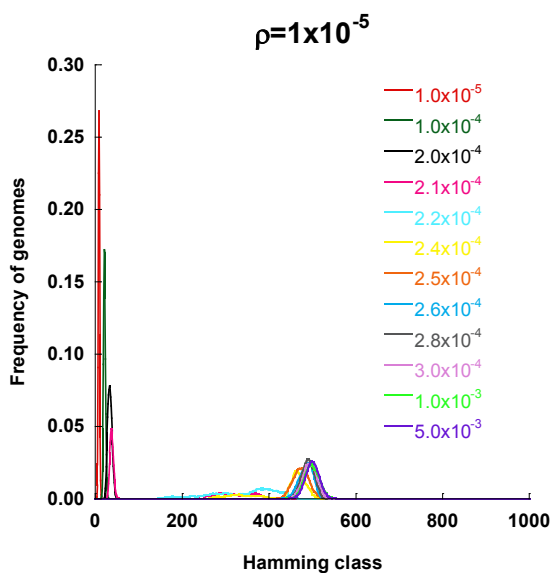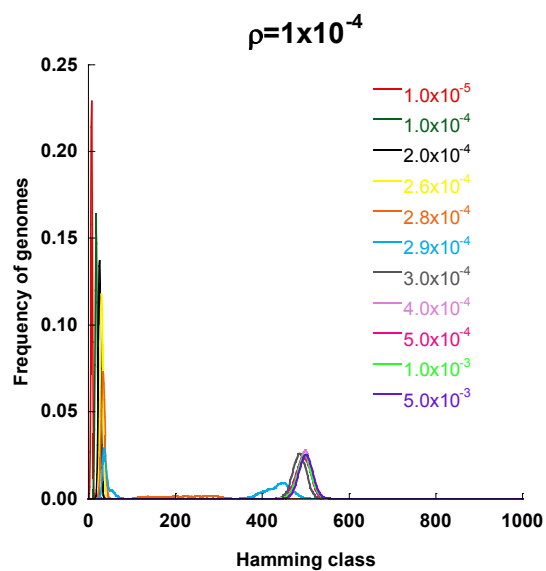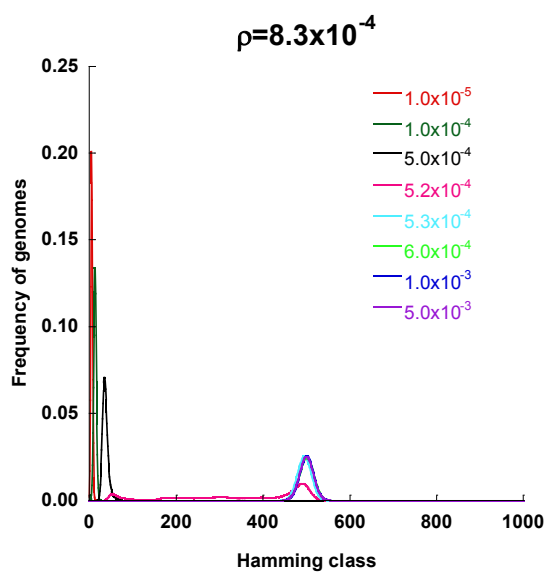

Supplement: Figure S5 — Quasispecies structure as a function of the recombination rate with M∼1 infection/cell. Structures of the quasispecies obtained with different recombination rates, (crossovers/site/replication), and over a range of values of (substitutions/site/replication) indicated, with M drawn from a distribution (Methods). (Some intermediate values of are omitted for clarity.) The corresponding steady state Shannon entropy, , and the resulting dependence of the error threshold, , on are presented in Figs. 6B and D. (PDF) [file pcbi.1002684.s005.pdf]

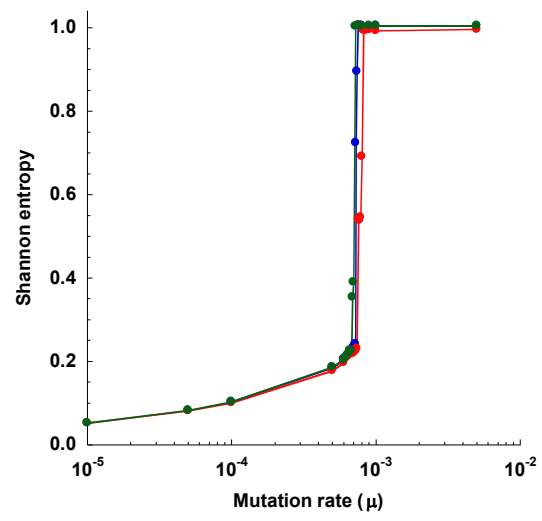

Supplement: Figure S6 — Dependence of the error threshold on the nucleotide composition of the founder sequence and nucleotide-specific mutation rates. The mean steady state Shannon entropy, , as a function of the mutation rate obtained with the founder sequence containing all nucleotides with equal frequencies and mutating at equal rates (blue), reproduced from Fig. 2C ( nucleotides). The corresponding when the founder sequence contained nucleotides at frequencies representative of HIV-1 (∼36% A's, 24% G's, 18% C's and 22% U's) mutating at equal rates (green) or at nucleotide-specific rates (, , , and ) (red). The other parameters are the same as in Fig. 2C. (PDF) [file pcbi.1002684.s006.pdf]

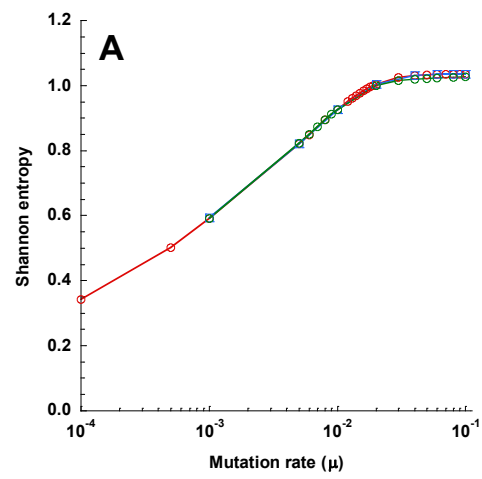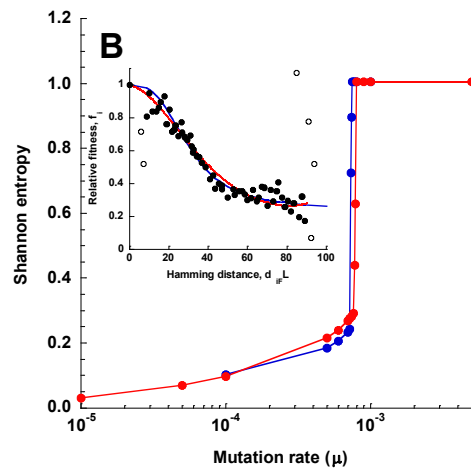

Supplement: Figure S7 — Dependence of the error threshold on the fitness landscape. (A) The mean steady state Shannon entropy, , as a function of the mutation rate obtained with the fitness landscape if and otherwise, with (red), 0.3 (blue), and 0.4 (green). Note that corresponds to the simulations in Fig. S1. With larger lengths, the fitness landscape has to be appropriately rescaled to avoid the extinction of the viral population due to severe fitness penalties (not shown). (B) as a function of obtained with the landscape (blue) and the polynomial fitness landscape (red). Note that the former data is the same as in Fig. 2C with nucleotides. Inset in (B) shows best fits of the two landscapes (blue and red lines, respectively) to data (symbols) excluding outliers (open symbols) from Bonhoeffer et al. (Science 306: 1547–1550 (2004)) modified to account for the observed frequencies of synonymous and non-synonymous mutations (see Balagam et al., PLoS ONE 6: e14531 (2011)). The best-fit parameter estimates are , and (blue); and , and (red). Because data was available only until Hamming distance ∼90 to which the polynomial can be fit, extrapolating the polynomial to higher Hamming distances yielded an unrealistic increase of fitness. To avoid this non-monotonic behavior, the fitness of genomes beyond the minimum (which occurred at Hamming distance 82) was set equal to the minimum. (PDF) [file pcbi.1002684.s007.pdf]

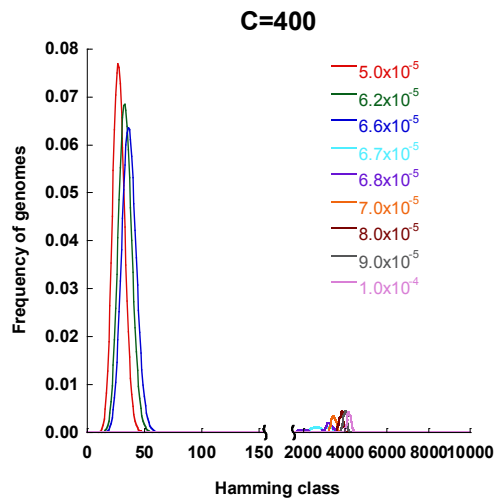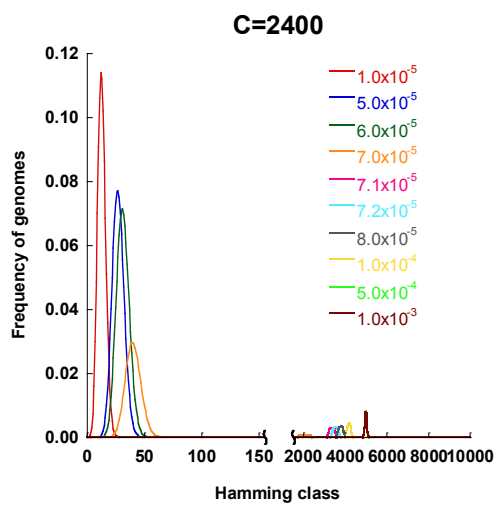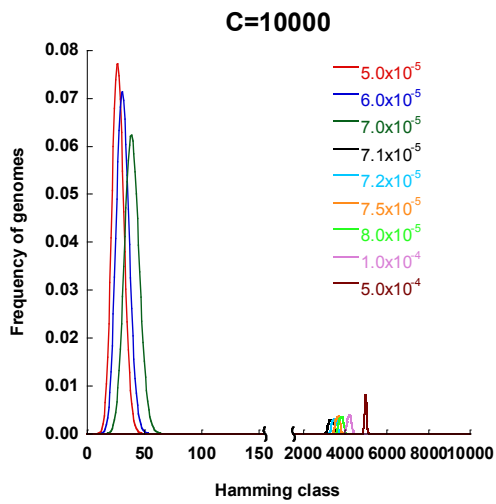

Supplement: Figure S8 — Quasispecies structures yielding estimates of the error threshold of HIV-1 with M = 3 infections/cell. Structures of the quasispecies obtained with different population sizes, C, and over a range of values of (substitutions/site/replication) indicated. (Some intermediate values of are omitted for clarity.) The corresponding dependence of the steady state Shannon entropy, , on is presented in Fig. 8A. (PDF) [file pcbi.1002684.s008.pdf]

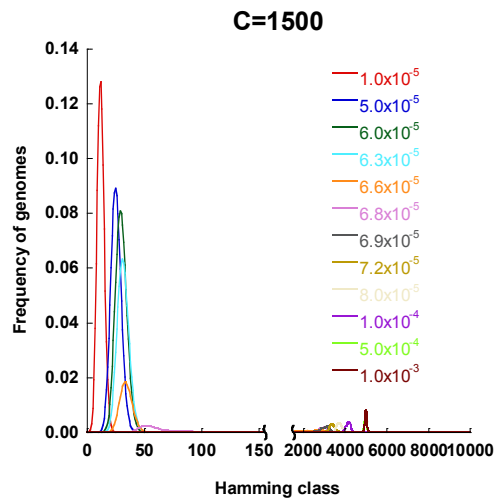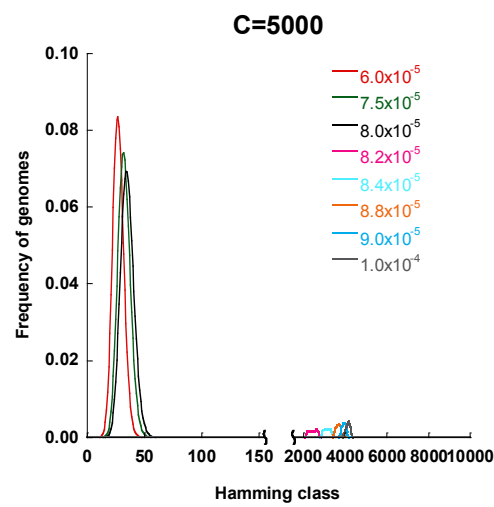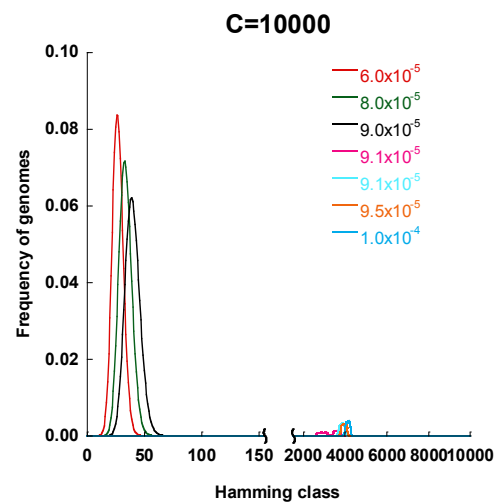

Supplement: Figure S9 — Quasispecies structures yielding estimates of the error threshold of HIV-1 with M∼1 infection/cell. Structures of the quasispecies obtained with different population sizes, C, and over a range of values of (substitutions/site/replication) indicated, with M drawn from a distribution (Methods). (Some intermediate values of are omitted for clarity.) The corresponding dependence of the steady state Shannon entropy, , on is presented in Fig. 8B. (PDF) [file pcbi.1002684.s009.pdf]
