# Supplementary material for: Stochastic Simulations Suggest that HIV-1 Survives Close to Its Error Threshold
Source: PLoS Comput Biol. 2012 Sep 13;8(9):e1002684. doi: 10.1371/journal.pcbi.1002684 (PMC3441496; doi:10.1371/journal.pcbi.1002684)
Supplement: Text S1 — The computer program employed for our simulations. (PDF) [file pcbi.1002684.s010.pdf]

```
/*
 * project: HIV quasispecies
 * file name: main.cc
 * date_created: 05-Dec-2010
 * date_modified: 10-Jul-2012
 * version: 1.3.0
*/

#include<stdio.h>
#include<cstdlib>
#include<math.h>
#include<iostream>
#include<time.h>
#include "mt19937-64.h" // Header file for Mersenne Twister random number generator

//#**** SIMULATION CONTROL ****#
// comment lines to switch on or off specific evolutionary processes
#define MUTATE_ON
#define RECOMBINE_ON
#define MULT_INF_ON //also change INF_DIST
#define DRIFT_ON
//#define FOUNDER_MUTATE_ON
#define FITNESS_SELECTION_ON

//#**** production and infection parameters ****#
#ifdef MULT_INF_ON
    #define MAXINF 3 // maximum number of infections per cell (define the specific distribution in next line)
    #define INF_DIST {0.0,0.0,100.0} // distribution of multiply infected cells
#else
    #define MAXINF 1
    #define INF_DIST {100.0}
#endif
#ifdef DRIFT_ON // multiple virions produced per cell
    #define MAXPRODUCE 10 // number of offspring viruses from each infected cell
#else
    #define MAXPRODUCE 1
#endif

//#**** simulation parameters ****#
#define MAXCYCLE 10000    // maximum number of generations
#define MAXRUNS 5 // maximum runs

//#**** system parameters ****#
#define L 10000      // Length of RNA sequence
#define MAXCELL 2400 // total number of cells
#define MAXVIRUS (MAXCELL*MAXPRODUCE) // total number of viruses

//#**** evolution parameters ****#
#ifdef MUTATE_ON // set specific mutation rate if MUTATE_ON switch is defined or set mutation rate to zero
    #define MUTATEINDEX 1.0e-3    // mutation rate (substitutions per site per replication)
#else
    #define MUTATEINDEX 0.0
#endif

#ifdef RECOMBINE_ON
    #define RECOMBINEINDEX 8.3e-4 // recombination rate (crossovers per site per replication)
#else
    #define RECOMBINEINDEX 0.0
#endif

#ifdef FOUNDER_MUTATE_ON
    #define FOUNDER_MUTATE_FRAC 0.1 // fraction of sites mutated from fittest sequence to form founder sequence
#else
    #define FOUNDER_MUTATE_FRAC 0.0
#endif

#ifdef FITNESS_SELECTION_ON   // calculate fitness of viruses if FITNESS_SELECTION_ON switch is defined or set virus fitness to 1.0
    #define VIRAL_FITNESS fitness(i)
#else
    #define VIRAL_FITNESS 1.0
#endif

```

```
//**** viral fitness parameters ****#
#define fMIN 0.2433  // minimum viral fitness
#define N 3.0    // Hill coefficient
#define d50 30.3 // Hamming distance for which viral fitness is half maximal

using namespace std;

double percnt_mult_inf[MAXINF] = INF_DIST;
int mult_inf[MAXINF] = {0};
int quasi_structure[L+1] = {0};
FILE *fp_quasi;

// virus class
class c_virus
  {
  public:
    int DNA[2*L]; // array containing two viral RNA sequences of length L
    double fitness; // fitness of virus
    bool available; // flag indicating whether virus is available for infection
    c_virus(); // virus constructor
  };

// virus class constructor (Default values when a virus is created)
c_virus::c_virus()
{
  for (int i=0;i<2*L;i++)
    {
      DNA[i] = 0; // set all nucleotides to A initially (0,1,2,3 corresponds to A, C, G and T respectively)
    }
  fitness = 1.0;
  available = true;
}

c_virus virus[MAXVIRUS]; // array containing viruses
c_virus fittest_virus;
c_virus founder_virus;

// T-cell class
class c_cell:public c_virus
  {
  public:
    c_virus V[MAXINF];   // array containing viruses that infected the cell
    int provirus[MAXINF][L]; // array for proviral DNA sequence
    int multinf; // present multiple infection status of cell

    void infect(int v,int inf); // function to infect the cell by virus with index number 'v' at multiple infection status 'inf'
    void mutate(); // function to mutate proviral sequence
    void recombine(); // function to create proviral sequence from viral RNA sequences by recombination
  };

c_cell cell[MAXCELL]; // array containing cells

// infect cell by virus with index number 'v' at multiple infection status 'inf'
void c_cell::infect(int v,int inf)
{
    // copy viral RNA sequence into cell
  for (int i=0;i<2*L;i++)
    {
      V[inf].DNA[i]=::virus[v].DNA[i];
    }

  multinf = multinf+1; // increase multiple infection status by 1
  //printf("multinf = %d ",multinf);
}//end of c_cell::infect()

// function to mutate proviral sequence
void c_cell::mutate()
{
  int ch_no = 2;
  // loop over present cell's multiple infection status (equal to number of proviral sequences)
  for(int i=0;i<multinf;i++)
    {
      // loop over proviral sequence length
```

```
      for (int j=0;j<L;j++)
        {
          // if random number is less than mutation rate change the nucleotide by 2 (A <-> G and C <-> T : only transitions are considered)
          if (rand_no()<MUTATEINDEX)
            {
              //printf("mutation occurred for provirus %d, position %d \n",i,j);
              provirus[i][j] = (provirus[i][j]+ch_no)%4;
            }
        }
    }
}//end of c_cell::mutate()


// function to create proviral DNA sequence from viral RNA sequence by recombination
void c_cell::recombine()
{
  // loop over present multiple infection status
  for (int i=0;i<multinf;i++)
    {
      int strand = 1;

      // select first or second strand randomly
      if (rand_no()>0.5)
        strand = 1;
      else
        strand = 2;

      // loop over proviral DNA sequence length
      for (int j=0;j<L;j++)
        {
          // if random number is less than recombination rate switch sequence
          if (rand_no() < RECOMBINEINDEX)
              {
                //printf("recombination occurred for provirus %d, position %d \n",i,j);
                  if (strand == 1)
                  strand = 2;
                    else
                      strand = 1;
               }

          // copy nucleotide from selected viral RNA strand to proviral DNA sequence
          if (strand == 1)
            {
              provirus[i][j] = V[i].DNA[j];
            }
          else
            {
              provirus[i][j] = V[i].DNA[L+j];
            }
        }
    }
}


int main()
{
  //int i,j,k;
  int tempv;
  int v1,v2;
  int vcount;
  int virus_prod_count = 0;

  double fittest_virus_fitness = 0.0; // fitness of fittest virus in current generation


  int time_now;
  time_t rawtime;
  struct tm * timeinfo;
  cout<<"Time of Start is"<<int(time(0))<<endl;   // simulation start time

  time_now = int(time(0));
  time( &rawtime);
  timeinfo = localtime ( &rawtime );
  cout<<"Date :"<<asctime(timeinfo); // simulation start date and time

  srand((long int)time(0)); // initialize random number generator
```

```
  void display_viral_sequences();
  void display_proviral_sequences();

  double fitness(int v); // function to calculate fitness
  void cal_quasi_structure(); // function to calculate quasispecies structure

  void initialize_virus(); // clear information in all viruses
  void initialize_cell(); // clear information in all cells
  void initialize_fittest_virus(); // create fittest virus
  void initialize_founder_virus(); // create founder virus

  // file pointer for simulation result summary
  FILE *fp_result;

  if ((fp_result = fopen("result.txt","a+"))==NULL)
    {
      printf("error in opening result.txt \n");
    }

  if ((fp_quasi = fopen("quasi_structure.txt","a+"))==NULL)
    {
      printf("error in opening quasi_structure.txt \n");
    }

  fprintf(fp_result,"%d\nSTART: %s\n",time_now,asctime(timeinfo));

  printf("L=%d, cell=%d, Maxruns=%d, Maxcycles=%d, Maxinf=%d, Maxprod=%d, mutateindex=%lf, recombine index=%lf, FOUNDER_MUTATE_FRAC=%lf \n",L,MAXCELL,MAXRUNS, MAXCYCLE, MAXINF, MAXPRODUCE, MUTATEINDEX, RECOMBINEINDEX,FOUNDER_MUTATE_FRAC);

  fprintf(fp_result,"L=%d, cell=%d, Maxruns=%d, Maxcycles=%d, Maxinf=%d, Maxprod=%d, mutateindex=%lf, recombine index=%lf, FOUNDER_MUTATE_FRAC=%lf \n",L,MAXCELL,MAXRUNS, MAXCYCLE, MAXINF, MAXPRODUCE, MUTATEINDEX, RECOMBINEINDEX,FOUNDER_MUTATE_FRAC);

  // display various switches' status
  #ifdef MUTATE_ON
    fprintf(fp_result,"MUTATION_ON\n");
    printf("MUTATION_ON\n");
  #endif
  #ifndef MUTATE_ON
    fprintf(fp_result,"MUTATION_OFF\n");
    printf("MUTATION_OFF\n");
  #endif

  #ifdef RECOMBINE_ON
    fprintf(fp_result,"RECOMBINATION_ON\n");
    printf("RECOMBINATION_ON\n");
  #endif
  #ifndef RECOMBINE_ON
    fprintf(fp_result,"RECOMBINATION_OFF\n");
    printf("RECOMBINATION_OFF\n");
  #endif

  #ifdef MULT_INF_ON
    fprintf(fp_result,"MULT_INF_ON\n");
    printf("MULT_INF_ON\n");
  #endif
  #ifndef MULT_INF_ON
    fprintf(fp_result,"MULT_INF_OFF\n");
    printf("MULT_INF_OFF\n");
  #endif

  #ifdef DRIFT_ON
    fprintf(fp_result,"DRIFT_ON\n");
    printf("DRIFT_ON\n");
  #endif
  #ifndef DRIFT_ON
    fprintf(fp_result,"DRIFT_OFF\n");
    printf("DRIFT_OFF\n");
  #endif

  #ifdef FOUNDER_MUTATE_ON
    fprintf(fp_result,"FOUNDER_MUTATE_ON\n");
    printf("FOUNDER_MUTATE_ON\n");
  #endif
  #ifndef FOUNDER_MUTATE_ON
```

```
    fprintf(fp_result,"FOUNDER_MUTATE_OFF\n");
    printf("FOUNDER_MUTATE_OFF\n");
  #endif

  #ifdef FITNESS_SELECTION_ON
    fprintf(fp_result,"FITNESS_SELECTION_ON\n");
    printf("FITNESS_SELECTION_ON\n");
  #endif
  #ifndef FITNESS_SELECTION_ON
    fprintf(fp_result,"FITNESS_SELECTION_OFF\n");
    printf("FITNESS_SELECTION_OFF\n");
  #endif

  /***********************************************/

  // calculate the number of cells of each multiple infected type from multiple infection distribution
  mult_inf[MAXINF-1] = MAXCELL;
  for(int i=0;i<MAXINF-1;i++)
  {
    mult_inf[i] = (int)rint(MAXCELL*percnt_mult_inf[i]/100);
    printf("mult_inf[%d]=%d \n",i,mult_inf[i]);
    mult_inf[MAXINF-1] = mult_inf[MAXINF-1]-mult_inf[i];
  }

    printf("mult_inf[%d]=%d \n",MAXINF-1,mult_inf[MAXINF-1]);

    // display multiple infection cell numbers
  for (int i=0;i<MAXINF;i++)
  {
    fprintf(fp_result,"mult_inf = %d \t percentage = %lf \t mult_inf_cells = %d\n",i+1, percnt_mult_inf[i],mult_inf[i]);
  }

  // loop over simulation runs
  for (int run=1;run<=MAXRUNS;run++)
    {
      init_genrand64((unsigned long long)(rand())); //initialize Mersenne Twister random number generator with a random number generated from GCC random number generator
      //printf("run =%d \n",run+1);

      /***** system initialization *****/
      initialize_virus();
      initialize_cell();

      initialize_fittest_virus(); // create fittest virus
      initialize_founder_virus(); // create founder virus

      /***** simulation start ******/
      // loop over generations
      for (int cycle=1;cycle<=MAXCYCLE;cycle++)
        {

          //printf("\n\n cycle start proviral sequences \n");
          //display_proviral_sequences();

          /*********** INFECTION **************/
          //printf("infecting \n");
           initialize_cell(); // clear all cell information to prepare for next round of infection
          int cell_count = 0;

          for (int i=1;i<=MAXINF;i++) // loop over multiple infected type
            {
              for(int j=0;j<mult_inf[i-1];j++) // loop over cells of each multiple infected type
              {
              for (int inf=0;inf<i;inf++) // loop over number of multiple infections in each cell
                {
                  do
                    {
                      tempv = (int)(rand_no()*MAXVIRUS); // select a virus randomly from surviving viral pool
                    }
                  while (virus[tempv].available==false);

```

```
        //printf("cell_count=%d ",cell_count);
        cell[cell_count].infect(tempv,inf); // infect the cell with the selected virus
        virus[tempv].available = false; // set infecting virus to false so that it is not available for further infections
        //printf("%d \n",tempv);
      }
    //printf("  ");
    cell_count++; // increment cell count
    }
  }
//printf("\n");

/*
 printf("cell multiple infection status \n");
for(int i=0;i<MAXCELL;i++)
{
  printf("%d ",cell[i].multinf);
}
printf("\n");
*/


 // set all viruses as not available, once infection process is complete
for (int i=0;i<MAXVIRUS;i++)
  {
    virus[i].available = false;
  }

/*********** RECOMBINATION & MUTATION ***********/
for (int i=0;i<MAXCELL;i++) // loop over all cells
  {
    //printf("cell=%d ",i);
    cell[i].recombine(); // perform recombination to produce proviral DNA from viral RNA
    cell[i].mutate(); // perform mutation on proviral DNA
  }

//printf("\n\nproviral sequences \n");
//display_proviral_sequences(); //after infection & reverse transcription


/********* PRODUCTION **********/
//printf("produced ");
virus_prod_count = 0;
for (int i=0;i<MAXCELL;i++) // loop over all cells
  {
    for (int prod=0;prod<MAXPRODUCE;prod++) // loop over viral production per cell
      {
        do
          {
           // select a virus position from viral pool to copy offspring virus
           // if the viral position is not already occupied
            tempv = (int)(rand_no()*MAXVIRUS);
          }
        while (virus[tempv].available == true);

         // select two proviral DNA strands existing inside cell randomly to copy into new offspring virus
        v1 = (int)(rand_no()*cell[i].multinf);
        v2 = (int)(rand_no()*cell[i].multinf);
        //printf("cell=%d,%d virus=%d provirus=%d,%d \n",i,prod,tempv,v1,v2);

         // copy proviral DNA strands to viral RNA strands
        for (int l=0;l<L;l++)
          {
            virus[tempv].DNA[l] = cell[i].provirus[v1][l];
            virus[tempv].DNA[L+l] = cell[i].provirus[v2][l];
          }
        virus[tempv].available = true; // set the virus as available for infection
        virus_prod_count++; // increment viral production count
        //printf("%d ",tempv);
      }
    //printf("  ");
  }
```

```
          //printf("\n\n");

          //printf("proviral sequences \n");
          //display_proviral_sequences();  //at the end of each cycle
          //printf("\n\n viral sequences \n");
          //display_viral_sequences();  //at the end of each cycle

          // display information regarding current generation and simulation run
      cout<<"run="<< run <<",cycle="<< cycle <<endl;
          //printf("run=%d cycle=%d \n",run,cycle);

         // calculate quasispecies structure of proviral DNA pool
      cal_quasi_structure();

          /*********** FITNESS SELECTION ********/
      fittest_virus_fitness = 0.0; // set fittest virus fitness to zero
          for (int i=0;i<MAXVIRUS;i++) // loop over all viruses
            {
              virus[i].fitness = VIRAL_FITNESS; //fitness(i); calculate virus fitness
              //printf("fitness of virus[%d]=%lf \n",i,virus[i].fitness);

               // if the virus fitness is larger than fittest virus fitness
               // set fittest virus fitness value to present virus fitness value
               if(fittest_virus_fitness < virus[i].fitness)
                 {
                   fittest_virus_fitness = virus[i].fitness;
                 }
            }

        vcount=0;

         // fitness selection of viruses
          for (int i=0;i<MAXVIRUS;i++) // loop over all viruses
            {
               // if random number is greater than viral fitness normalized by the fittest virus fitness
               // then set present virus as not available for infection
              if (rand_no()>(virus[i].fitness/fittest_virus_fitness))
                {
                  //printf("virus %d not available \n",i);
                  virus[i].available = false;
                  vcount++;
                }
            }

          cout<<"no of viruses surviving "<<(virus_prod_count-vcount)<<endl;
          //printf("no of viruses surviving %d \n",(MAXVIRUS-vcount));

      // flush file buffers for immediate data writing to file
      fflush(fp_result);
      fflush(fp_quasi);
        } /***** end of cycle *****/

      //display_viral_sequences();  //at the end of each run

    } /***** end of runs *****/

  // Time of simulation end
  cout<<"Time of Ending is"<<int(time(0))<<endl;
  time( &rawtime);
  timeinfo = localtime ( &rawtime );

  cout<<"Date :"<<asctime(timeinfo);  // date and time at the end of simulation
  fprintf(fp_result,"\n\n%d\nEND: %s \n",int(time(0)),asctime(timeinfo));


  // close all simulation result files
  fclose(fp_result);
  fclose(fp_quasi);

  return(0);
}//end of main
/**********************************************************/
```

```
void display_viral_sequences()
{
 for (int i=0;i<MAXVIRUS;i++)
  {
   for (int j=0;j<2*L;j++)
    {
     printf("%d\t",virus[i].DNA[j]);
    }
   printf("\n");
  }
}

void display_proviral_sequences()
{
 for (int i=0;i<MAXCELL;i++)
  {
   //for (int j=0;j<MAXINF;j++)
   for (int j=0;j<cell[i].multinf;j++)
    {
     printf("cell=%d provirus=%d\n",i,j);
     for (int l=0;l<L;l++)
      {
       printf("%d\t",cell[i].provirus[j][l]);
      }
     printf("\n");
    }
   printf("\n");
  }
}

// calculate quasispecies structure
void cal_quasi_structure()
{
 int hamming = 0;
 long int nprovirus = 0;
 long int count = 0;

 for(int l=0;l<=L;l++)
  {
   quasi_structure[l] = 0;
  }

 for (int i=0;i<MAXCELL;i++) // loop over all cells
  {
   for(int j=0;j<cell[i].multinf;j++) // loop over number of provirues in each cell
    {
     count++;
     hamming = 0;
     for (int k=0;k<L;k++) // loop over proviral DNA length
      {
       // calculate number of differences between fittest virus
       // and present provirus for classification into hamming classes
     if (cell[i].provirus[j][k] != fittest_virus.DNA[k])
        hamming++;
        //printf("hamming = %d \n",hamming);
      }
       // increment hamming class count by 1 in quasispecies structure
     quasi_structure[hamming] = quasi_structure[hamming] + 1;
    }
  }

  // sum to calculate total number of proviruses in all cells
 for(int inf=0;inf<MAXINF;inf++)
  {
   nprovirus = nprovirus + mult_inf[inf]*(inf+1);
  }

 //printf("nprovirus = %d count = %d\n",nprovirus,count);

 // store quasispecies structure into file
 for(int len=0;len<=L;len++)
  {
   fprintf(fp_quasi,"%d \t",quasi_structure[len]);
   //printf("%d \t",quasi_structure[len]);
  }
 fprintf(fp_quasi,"\n");
```

```
}

// set all viral nucleotides to A for all viruses
void initialize_virus()
{
  for (int i=0;i<MAXVIRUS;i++)
    {
      for (int j=0;j<2*L;j++)
        {
          virus[i].DNA[j] = 0;
        }
    }
}

// create fittest virus
void initialize_fittest_virus()
{
  double rand = 0.0;

  // select each nucleotide position uniformly from A, G, C and T
  for (int k=0;k<L;k++)
    {
        rand = rand_no();

      if (rand < 0.25)
        {
          fittest_virus.DNA[k] = 0;
          fittest_virus.DNA[L+k] = 0;
        }
      else if (rand < 0.50)
        {
          fittest_virus.DNA[k] = 1;
          fittest_virus.DNA[L+k] = 1;
        }
      else if (rand < 0.75)
        {
          fittest_virus.DNA[k] = 2;
          fittest_virus.DNA[L+k] = 2;
        }
      else
        {
          fittest_virus.DNA[k] = 3;
          fittest_virus.DNA[L+k] = 3;
        }
    }

  fittest_virus.fitness = 1.0;
}

// create founder virus
void initialize_founder_virus()
{
    long int hamming = 0;
    int ch_no = 2;
    int rand_pos;

    for(int j=0;j<L;j++) //copying of fittest virus to founder virus
    {
      founder_virus.DNA[j] = fittest_virus.DNA[j];
      founder_virus.DNA[L+j] = fittest_virus.DNA[L+j];
    }

    /* mutating fraction of first virus genome length */
    for (int count=0;count<(L*FOUNDER_MUTATE_FRAC);)
     {
       rand_pos = (int)(rand_no()*L);
       //cout<<rand_pos<<" ";
       if(founder_virus.DNA[rand_pos] != fittest_virus.DNA[rand_pos])
       continue;
       else
        {
          founder_virus.DNA[rand_pos] = (fittest_virus.DNA[rand_pos]+ch_no)%4;
          founder_virus.DNA[L+rand_pos] = (fittest_virus.DNA[L+rand_pos]+ch_no)%4;
          count++;
        }
     }

```

```
    /* founder virus fitness calculation */
    for(int i=0;i<2*L;i++)
    {
        if (founder_virus.DNA[i] != fittest_virus.DNA[i])
        hamming++;
    }

    hamming = (long int)(hamming/2.0);

    founder_virus.fitness = 1.0 - (1.0-fMIN)*pow(hamming,N)/(pow(hamming,N) + pow(d50,N));

   /* copy founder_virus to initial viral pool */
    for (int i=0;i<MAXVIRUS;i++)
    {
        for(int j=0;j<2*L;j++)
        {
            virus[i].DNA[j] = founder_virus.DNA[j];
        }

        virus[i].fitness = founder_virus.fitness;
        virus[i].available = true;
    }
}


// set all viral and proviral sequences to zero in all cells
void initialize_cell()
{
  for (int i=0;i<MAXCELL;i++)
    {
      for (int j=0;j<MAXINF;j++)
        {
          for (int k=0;k<L;k++)
            {
              cell[i].V[j].DNA[k] = 0;
              cell[i].V[j].DNA[L+k] = 0;
              cell[i].provirus[j][k] = 0;
            }
        }
        cell[i].multinf = 0;
    }
}

// calculate fitness of virus with index number 'v'
double fitness(int v)
{
  long int hamming = 0;
  double fitness = 0.0;

  for (int i=0;i<2*L;i++)
    {
      if (virus[v].DNA[i] != fittest_virus.DNA[i])
          hamming++;
    }

  hamming = (long int)(hamming/2.0);

  fitness = 1.0 - (1.0-fMIN)*pow(hamming,N)/(pow(hamming,N) + pow(d50,N));

  //printf("hamming value = %d fitness = %lf \n",hamming,fitness);
  return(fitness);
}
```

```c
/*
 * project: HIV quasispecies
 * file name: mt19937-64.h
 * date: 31-10-08
 * version: 9.2
 * remarks: changed the name of the function "genrand64_real2(void)" to "rand_no(void)"
 */

#ifndef MT1993764_H_
#define MT1993764_H_

/*
   A C-program for MT19937-64 (2004/9/29 version).
   Coded by Takuji Nishimura and Makoto Matsumoto.

   This is a 64-bit version of Mersenne Twister pseudorandom number
   generator.

   Before using, initialize the state by using init_genrand64(seed)
   or init_by_array64(init_key, key_length).

   Copyright (C) 2004, Makoto Matsumoto and Takuji Nishimura,
   All rights reserved.

   Redistribution and use in source and binary forms, with or without
   modification, are permitted provided that the following conditions
   are met:

     1. Redistributions of source code must retain the above copyright
        notice, this list of conditions and the following disclaimer.

     2. Redistributions in binary form must reproduce the above copyright
        notice, this list of conditions and the following disclaimer in the
        documentation and/or other materials provided with the distribution.

     3. The names of its contributors may not be used to endorse or promote
        products derived from this software without specific prior written
        permission.

   THIS SOFTWARE IS PROVIDED BY THE COPYRIGHT HOLDERS AND CONTRIBUTORS
   "AS IS" AND ANY EXPRESS OR IMPLIED WARRANTIES, INCLUDING, BUT NOT
   LIMITED TO, THE IMPLIED WARRANTIES OF MERCHANTABILITY AND FITNESS FOR
   A PARTICULAR PURPOSE ARE DISCLAIMED.  IN NO EVENT SHALL THE COPYRIGHT OWNER OR
   CONTRIBUTORS BE LIABLE FOR ANY DIRECT, INDIRECT, INCIDENTAL, SPECIAL,
   EXEMPLARY, OR CONSEQUENTIAL DAMAGES (INCLUDING, BUT NOT LIMITED TO,
   PROCUREMENT OF SUBSTITUTE GOODS OR SERVICES; LOSS OF USE, DATA, OR
   PROFITS; OR BUSINESS INTERRUPTION) HOWEVER CAUSED AND ON ANY THEORY OF
   LIABILITY, WHETHER IN CONTRACT, STRICT LIABILITY, OR TORT (INCLUDING
   NEGLIGENCE OR OTHERWISE) ARISING IN ANY WAY OUT OF THE USE OF THIS
   SOFTWARE, EVEN IF ADVISED OF THE POSSIBILITY OF SUCH DAMAGE.

   References:
   T. Nishimura, ``Tables of 64-bit Mersenne Twisters''
     ACM Transactions on Modeling and
     Computer Simulation 10. (2000) 348--357.
   M. Matsumoto and T. Nishimura,
     ``Mersenne Twister: a 623-dimensionally equidistributed
       uniform pseudorandom number generator''
     ACM Transactions on Modeling and
     Computer Simulation 8. (Jan. 1998) 3--30.

   Any feedback is very welcome.
   http://www.math.hiroshima-u.ac.jp/~m-mat/MT/emt.html
   email: m-mat @ math.sci.hiroshima-u.ac.jp (remove spaces)
*/


#include <stdio.h>

#define NN 312
#define MM 156
#define MATRIX_A 0xB5026F5AA96619E9ULL
#define UM 0xFFFFFFFF80000000ULL /* Most significant 33 bits */
#define LM 0x7FFFFFFFULL /* Least significant 31 bits */


/* The array for the state vector */
```

```c
static unsigned long long mt[NN];
/* mti==NN+1 means mt[NN] is not initialized */
static int mti=NN+1;

/* initializes mt[NN] with a seed */
void init_genrand64(unsigned long long seed)
{
    mt[0] = seed;
    for (mti=1; mti<NN; mti++)
        mt[mti] =  (6364136223846793005ULL * (mt[mti-1] ^ (mt[mti-1] >> 62)) + mti);
}

/* initialize by an array with array-length */
/* init_key is the array for initializing keys */
/* key_length is its length */
//unsigned long long init_key[], key_length;
void init_by_array64(unsigned long long init_key[], unsigned long long key_length)
{
    unsigned long long i, j, k;
    init_genrand64(19650218ULL);
    i=1; j=0;
    k = (NN>key_length ? NN : key_length);
    for (; k; k--) {
        mt[i] = (mt[i] ^ ((mt[i-1] ^ (mt[i-1] >> 62)) * 3935559000370003845ULL))
          + init_key[j] + j; /* non linear */
        i++; j++;
        if (i>=NN) { mt[0] = mt[NN-1]; i=1; }
        if (j>=key_length) j=0;
    }
    for (k=NN-1; k; k--) {
        mt[i] = (mt[i] ^ ((mt[i-1] ^ (mt[i-1] >> 62)) * 2862933555777941757ULL))
          - i; /* non linear */
        i++;
        if (i>=NN) { mt[0] = mt[NN-1]; i=1; }
    }

    mt[0] = 1ULL << 63; /* MSB is 1; assuring non-zero initial array */
}

/* generates a random number on [0, 2^64-1]-interval */
unsigned long long genrand64_int64(void)
{
    int i;
    unsigned long long x;
    static unsigned long long mag01[2]={0ULL, MATRIX_A};

    if (mti >= NN) { /* generate NN words at one time */

        /* if init_genrand64() has not been called, */
        /* a default initial seed is used     */
        if (mti == NN+1)
            init_genrand64(5489ULL);

        for (i=0;i<NN-MM;i++) {
            x = (mt[i]&UM)|(mt[i+1]&LM);
            mt[i] = mt[i+MM] ^ (x>>1) ^ mag01[(int)(x&1ULL)];
        }
        for (;i<NN-1;i++) {
            x = (mt[i]&UM)|(mt[i+1]&LM);
            mt[i] = mt[i+(MM-NN)] ^ (x>>1) ^ mag01[(int)(x&1ULL)];
        }
        x = (mt[NN-1]&UM)|(mt[0]&LM);
        mt[NN-1] = mt[MM-1] ^ (x>>1) ^ mag01[(int)(x&1ULL)];

        mti = 0;
    }

    x = mt[mti++];

    x ^= (x >> 29) & 0x5555555555555555ULL;
    x ^= (x << 17) & 0x71D67FFFEDA60000ULL;
    x ^= (x << 37) & 0xFFF7EEE000000000ULL;
    x ^= (x >> 43);

    return x;
}
```

```
/* generates a random number on [0, 2^63-1]-interval */
long long genrand64_int63(void)
{
    return (long long)(genrand64_int64() >> 1);
}

/* generates a random number on [0,1]-real-interval */
double genrand64_real1(void)
{
    return (genrand64_int64() >> 11) * (1.0/9007199254740991.0);
}

/* generates a random number on [0,1)-real-interval */
//double genrand64_real2(void) ( ********* modification to function name ************)
double rand_no(void)
{
    return (genrand64_int64() >> 11) * (1.0/9007199254740992.0);
}

/* generates a random number on (0,1)-real-interval */
double genrand64_real3(void)
{
    return ((genrand64_int64() >> 12) + 0.5) * (1.0/4503599627370496.0);
}

/*
int main(void)
{
    int i;
    unsigned long long init[4]={0x12345ULL, 0x23456ULL, 0x34567ULL, 0x45678ULL}, length=4;
    init_by_array64(init, length);
    printf("1000 outputs of genrand64_int64()\n");
    for (i=0; i<1000; i++) {
      printf("%20llu ", genrand64_int64());
      if (i%5==4) printf("\n");
    }
    printf("\n1000 outputs of genrand64_real2()\n");
    for (i=0; i<1000; i++) {
      printf("%10.8f ", genrand64_real2());
      if (i%5==4) printf("\n");
    }
    return 0;
}

*/

#endif /*MT1993764_H_*/
```
